# Induction of immunosuppressive functions and NF-κB by FLIP in monocytes

**DOI:** 10.1038/s41467-018-07654-4

**Published:** 2018-12-05

**Authors:** Alessandra Fiore, Stefano Ugel, Francesco De Sanctis, Sara Sandri, Giulio Fracasso, Rosalinda Trovato, Silvia Sartoris, Samantha Solito, Susanna Mandruzzato, Fulvia Vascotto, Keli L. Hippen, Giada Mondanelli, Ursula Grohmann, Geny Piro, Carmine Carbone, Davide Melisi, Rita T. Lawlor, Aldo Scarpa, Alessia Lamolinara, Manuela Iezzi, Matteo Fassan, Silvio Bicciato, Bruce R. Blazar, Ugur Sahin, Peter J. Murray, Vincenzo Bronte

**Affiliations:** 1 Department of Medicine, Section of Immunology, University of Verona, Verona, 37134 Italy; 2 Department of Surgery, Oncology and Gastroenterology, Section of Oncology and Immunology, University of Padova, Padova, 35124 Italy; 3 Istituto Oncologico Veneto IOV-IRCCS, Padova, 35124 Italy; 4 TRON-Translational Oncology, University Medical Center of Johannes Gutenberg University, Mainz, 55131 Germany; 5 Department of Pediatrics, Division of Blood and Marrow Transplantation, University of Minnesota, Minneapolis, 55455 MN USA; 6 Department of Experimental Medicine, University of Perugia, Perugia, 06132 Italy; 7 Department of Medicine, Digestive Molecular Clinical Oncology Research Unit, University of Verona, Verona, 37134 Italy; 8 Department of Medicine, Laboratory of Oncology and Molecular Therapy, University of Verona, Verona, 37134 Italy; 9 ARC-Net Centre for Applied Research on Cancer, University and Hospital Trust of Verona, Verona, 37134 Italy; 10 Department of Pathology and Diagnostics, University of Verona, Verona, 37134 Italy; 11 Department of Medicine and Aging Science, Center of Excellence on Aging and Translational Medicine (CeSi-Met), University G. D’Annunzio of Chieti-Pescara, Chieti, 66100 Italy; 12 Department of Medicine-DIMED, University of Padova, Padova, 35124 Italy; 13 Department of Life Sciences, Center for Genome Research, University of Modena and Reggio Emilia, Modena, 41100 Italy; 14 University Medical Center of the Johannes Gutenberg University, Mainz, 55131 Germany; 15 Biopharmaceutical New Technologies (BioNTech) Corporation, Mainz, 55131 Germany; 16 Max Planck Institute of Biochemistry, Martinsried, 82152 Germany; 17 Present Address: Max Planck Institute of Biochemistry, Martinsried, 82152 Germany

## Abstract

Immunosuppression is a hallmark of tumor progression, and treatments that inhibit or deplete monocytic myeloid-derived suppressive cells could promote anti-tumor immunity. c-FLIP is a central regulator of caspase-8-mediated apoptosis and necroptosis. Here we show that low-dose cytotoxic chemotherapy agents cause apoptosis linked to c-FLIP down-regulation selectively in monocytes. Enforced expression of c-FLIP or viral FLIP rescues monocytes from cytotoxicity and concurrently induces potent immunosuppressive activity, in T cell cultures and in vivo models of tumor progression and immunotherapy. FLIP-transduced human blood monocytes can suppress graft versus host disease. Neither expression of FLIP in granulocytes nor expression of other anti-apoptotic genes in monocytes conferred immunosuppression, suggesting that FLIP effects on immunosuppression are specific to monocytic lineage and distinct from death inhibition. Mechanistically, FLIP controls a broad transcriptional program, partially by NF-κB activation. Therefore, modulation of FLIP in monocytes offers a means to elicit or block immunosuppressive myeloid cells.

## Abstract (web summary)

Signaling and transcriptional regulation of MDSC activity remains largely undefined. Here the authors show that monocytic MDSC immunosuppression is triggered by c-FLIP and requires NFκB, implicate this axis in cancer prognosis and response to therapy, and employ ectopic FLIP to treat immunopathology.

## Introduction

The current treatments for cancer patients rely on cytotoxic agents able to destroy malignant cells^1^ that have acquired distinctive chronic proliferation by evading from cell death checkpoints, as well as by self-generating proliferative signals^2^. At the same time, chemotherapy can cause systemic immune modulation at multiple levels^3,4^. For example, some chemotherapeutics induce immune depression by favoring myelo- and lympho-penia^5^; on the other hand, chemotherapeutic drugs can exert immune stimulatory actions by favoring the activation of anti-tumor T cells, both through the induction of immunogenic tumor cell death^3,6^ and containment of immunosuppressive immune cell populations, such as regulatory T cells (Treg) and myeloid-derived suppressor cells (MDSCs)^7,8^. Chemotherapy can thus be used to restore immune responses in tumor-bearing hosts. Certain pharmacologically active substances can eliminate monocytic (M)-MDSCs in different preclinical models^9^ and carboplatin and paclitaxel normalized myeloid cell numbers in advanced cervical cancer patients, increasing the response to a peptide-based vaccine^8^. Considering the many unwanted side effects of chemotherapy, however, definition of the intracellular targets accounting for the exquisite activity of different chemotherapeutics on M-MDSCs is needed for focused molecular approaches. For instance, monocyte/macrophage depletion by trabectedin depends on increased levels of membrane death receptors (Fas and tumor necrosis factor-related apoptosis inducing ligand [TRAIL] receptor 2) that facilitate the recruitment of caspase-8 and the activation of the apoptotic cascade^10^. However, this biological modulation might not be shared by other drugs.

The major player in TRAIL-induced apoptosis resistance is cellular FLICE (FADD-like IL-1β-converting enzyme)-inhibitory protein (c-FLIP)^11^. The gene encoding c-FLIP (*CFLAR*) is conserved in vertebrates and structurally similar to viral FLICE-inhibitory protein (v-FLIP) that is encoded in the genome of gamma herpesviruses such as the human herpesvirus 8, the Kaposi’s sarcoma-associated virus^12,13^. It is activated by several stimuli, such as growth factors, tumor necrosis factor (TNF)-ligands and chemotherapy and it is transcriptionally regulated, especially by nuclear factor kappa-light-chain-enhancer of activated B cells (NF-κB)^14^. In turn, c-FLIP regulates important processes maintaining immune system homeostasis: it protects mature T lymphocytes from activation-induced cell death^15^, it controls Treg homeostasis^16^ and survival from FasL-mediating killing by tumor endothelium^17^, as well as Fas sensitivity of monocytes and monocyte-derived cells, such as dendritic cells (DCs) and macrophages^18,19^ and their viability in the bone marrow during normal development^20^. Recently, c-FLIP expression was shown to represent *a conditio sine qua non* for tumor-induced, M-MDSC generation^21^.

FLIP expression might buy time for myeloid cells and protect monocytes and macrophages, allowing them to perform their functions in a hostile inflammatory environment. This is likely the case for cancer^21^ but it also applies to lung macrophages during post-damage fibrosis^22^; moreover, FLIP can also limit the negative consequences of caspase-8 activation by inflammasome sensors in macrophages^23^. Thus, FLIP may have acquired other properties during the evolution, contributing more directly to dampen the inflammation in a monocyte/macrophage extrinsic fashion. Here we report a dual role of FLIP in myeloid cells. We found that drugs able to restrain FLIP expression selectively eliminate M-MDSCs but not polymorphonuclear (PMN)-MDSCs restoring T cell responses; more importantly, expression of FLIP in human normal myeloid precursors and monocytes is sufficient to confer the immune suppressive properties associated with MDSCs.

## Results

### c-FLIP protects M-MDSCs from chemotherapy-induced killing

**Fig. 1:**
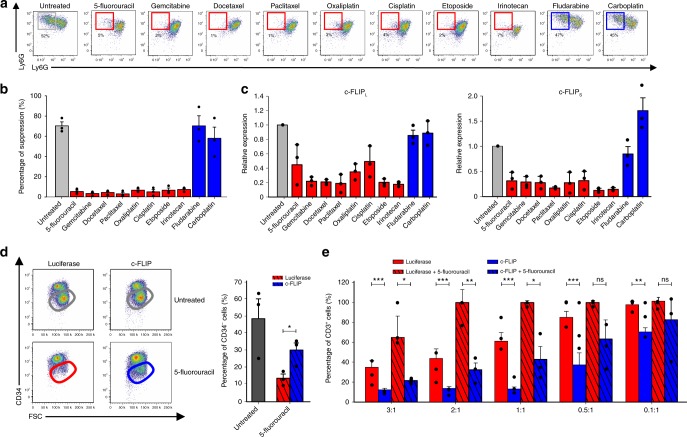
c-FLIP protects in vitro generated M-MDSCs from chemotherapy-induced death. **a** Representative flow cytometry plots of mouse BM-MDSCs after chemotherapy treatment. Untreated Ly6C^+^ cells are in black squares; in red and in blue, Ly6C^+^ cells after exposure to the highest dose of chemotherapy that maintains the cell viability (defined as ≥75% of AnnV^-^/7AAD^-^ cells). **b** Suppressive activity of untreated (black bar) or chemotherapy-treated BM-MDSCs (red bars indicate M-MDSC-affecting chemotherapeutics whereas blue bars indicate ineffective chemotherapeutics) was measured by CellTrace dilution in peptide-activated CD8^+^ T cells. **c** Expression levels of both c-FLIP_L_ and c-FLIP_S_ in Ly6C^+^ cells purified from BM-MDSCs treated with different chemotherapy were evaluated by real-time PCR. **d** Flow cytometry analysis of c-FLIP infected human in vitro generated BM-MDSCs (identified as CD34^-^ cells) after 5-fluorouracil treatment. **e** Functional assay on luciferase- and c-FLIP-infected BM-MDSCs derived from in vitro differentiation of hCD34^+^ cells, either left untreated or after exposure to a M-MDSC-affecting dose of 5-fluorouracil. Data are presented either as mean ± s.e.m of three independent experiments (**b**–**e**) or as a representative experiment of three independent experiments (**a**). *P < 0.05, **P < 0.01; ***P < 0.001; n.s., not significant, by Mann–Whitney test

We previously reported that low doses of diverse chemotherapeutic drugs, which are unable to control tumor growth, selectively affect the numbers of circulating CD11b^+^Ly6G^−^Ly6C^high^ cells and enhance the efficacy of adoptive cell therapy (ACT)^9^. To understand the molecular basis of this differential susceptibility, we compared 10 conventional anti-cancer drugs to test their ability to modulate in vitro CD11b^+^Ly6G^−^Ly6C^high^ cell viability during bone marrow (BM)-MDSC differentiation^24^. After testing different doses of each drug, we defined the highest drug concentration that did not cause overt toxicity, i.e. ≥ 75% of cells were viable at the end of culture (Supplementary Fig. 1a, b). Except for fludarabine and carboplatin, the addition of all the tested chemotherapeutics caused a redistribution within the myeloid subsets (Fig. 1a and Supplementary Fig. 1c), characterized by a contraction in CD11b^+^Ly6G^−^Ly6C^high^ cells (M-MDSCs) while sparing CD11b^+^Ly6G^+^Ly6C^low/int^ cells (PMN-MDSCs). Furthermore, only those drugs effective in reducing M-MDSCs eliminated the immune suppressive activity of cultured cells on activated T lymphocytes (Fig. 1b). With a dose-response curve similar to mouse BM-MDSCs, also human CD11b^bright^ cells, which mostly contain PMN-MDSCs, obtained by in vitro culture from bone marrow (BM) Lin^−^ cells in the presence of G-CSF and GM-CSF for four days^25^, survived after exposure to different chemotherapeutics (Supplementary Fig. 1d, e) at the expense of other fractions and this led to loss of immune suppressive activity, as shown for the drug 5-fluorouracil (Supplementary Fig. 1f).

To shed light on the possible mechanisms underlying the cytotoxic action of the diverse chemotherapeutic drugs, we treated MSC2 cells, an immortalized mouse MDSC line^26^, with the minimal effective dose of one agent for each class. All tested drugs activated caspases associated with extrinsic apoptotic pathways, with similar but not overlapping kinetics (Supplementary Fig. 1g). Moreover, only the addition of a pan-caspase inhibitor to 5-fluorouracil-treated MSC2 cells restored cell viability, whereas no rescue was achieved with a necroptosis inhibitor (Supplementary Fig. 1h). Considering the role of c-FLIP in the survival of M-MDSCs but not PMN-MDSCs^21^, we speculated that chemotherapeutics might modulate this anti-apoptotic protein. Indeed, Ly6C^+^ cells purified from BM-MDSC cultures after a 24 h treatment with M-MDSC-depleting drugs downregulated the main c-FLIP isoforms, whereas drugs unable to deplete M-MDSCs did not affect c-FLIP RNA expression (Fig. 1c). These observations provide evidence for a correlation between FLIP expression and chemotherapy susceptibility in monocytes.

We next verified whether c-FLIP was associated with chemotherapy resistance in human MDSCs. We modified a previously described protocol^25^ to generate immunosuppressive MDSCs, which could be infected in vitro by lentiviruses, by culturing BM-derived stem cells (CD34^+^ cells) for four days in the presence of G-CSF and GM-CSF. After the in vitro culture, CD34^+^ cells differentiated into mature myeloid cells characterized by the downregulation of the CD34 marker and acquisition of myeloid lineage markers (CD33, CD11b, CD15, and CD14) (Supplementary Fig. 2a). During differentiation, cells also acquired immunosuppressive functions typical of MDSCs^27^ (Supplementary Fig. 2b). We next infected human CD34^+^ cells with lentiviral expression vectors encoding the two major splice variants of human c-FLIP or luciferase, as negative control, and cultured them in G-CSF and GM-CSF in presence of 5-fluorouracil. The enforced expression of c-FLIP prevented chemotherapy-dependent killing of more mature myeloid cells (CD34^−^ cells, Fig. 1d) and preserved their immunosuppressive activity since 5-fluorouracil-treated, c-FLIP-infected cells maintained their ability to arrest in vitro T cell proliferation compared to the control (Fig. 1d).

These data indicate that chemotherapy-driven elimination of human MDSCs is dependent on c-FLIP modulation. Furthermore, compared to luciferase-expressing cells, untreated c-FLIP-expressing CD34^+^ cells showed higher suppressive activity toward activated T cells, which was still evident at inhibitor to effector cell ratio as low as 0.1:1 (Fig. 1e). Therefore, c-FLIP not only controlled myeloid cell survival but also modulated their immune regulatory ability.

### v-FLIP^+^ monocytes are chemotherapy-resistant and tolerogenic

**Fig. 2:**
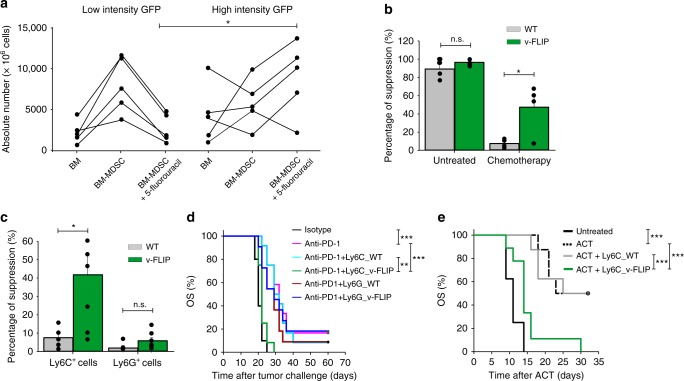
v-FLIP^+^Ly6C^+^ cells are chemotherapy-resistant and immunosuppressive. **a** Absolute numbers of CD11b^+^Ly6C^+^ cells isolated from Tg mice in fresh BM, in vitro differentiated BM-MDSCs and in vitro differentiated, 5-fluorouracil-treated BM-MDSCs. **b** Suppressive activity of BM-MDSCs derived from 5-fluorouracil-treated BM of Tg mice was measured using peptide-activated CD8^+^ T cells by flow cytometry. **c** Suppressive activity of monocytic (CD11b^+^Ly6G^-^Ly6C^high^ cells) and PMN (CD11b^+^Ly6G^+^Ly6C^low^ cells) myeloid subsets freshly isolated from the BM of WT and Tg mice were measured using CellTrace-labeled OT-I cells. **d** Kaplan–Meier survival analysis of MCA205 tumor-bearing mice (*n* = 12 mice/group) treated with anti-PD-1 mAb (pink line) or isotype Ab (black line) using 4 intraperitoneal administrations every 2 days. Three days from the last treatment, some mice received the adoptive transfer of myeloid cells freshly isolated from the BM of either Tg or WT mice: v-FLIP-expressing Ly6C^+^ (green line), v-FLIP-expressing Ly6G^+^ cells (dark blue line), Ly6C^+^ cells (light blue line) or Ly6G^+^ cells (dark red line). **e** Kaplan–Meier survival analysis of EG7 tumor-bearing mice (*n* = 5 mice/group) were injected intravenously (i.v.) with 10^6^ OVA-specific OT-I T lymphocytes (ACT) and, after 2 h, mice were infused i.v. with 0.5 × 10^6^ CD11b^+^Ly6C^+^ fresh monocytes isolated from either WT or Tg mice. Data are presented either as mean ± s.e.m. of three independent experiments (**b**, **c**) or as two cumulated experiments (**d**) or as a representative experiment (**e**). *P < 0.05, **P < 0.01; ***P < 0.001; n.s., not significant, by Mann–Whitney test (**a**–**c**) and by log-rank test (**d**, **e**)

To dissect how FLIP proteins regulate myeloid cells we next took advantage of a transgenic (Tg) mouse expressing a form of FLIP from Kaposi’s sarcoma virus (hereafter called v-FLIP)^28^. We crossed ROSA26.vFLIP knock-in mice with mice expressing Cre recombinase under the control of the endogenous *Lyz2* promoter, thus resulting in mice with v-FLIP expression in the myeloid lineage. In these mice, EGFP is expressed in a common transcript with v-FLIP due to the insertion of an IRES between the two gene sequences^28^. Tg mice (carrying both Cre and v-FLIP genes) were born at the expected Mendelian frequency but showed developmental abnormalities and all the mice died within four weeks (Supplementary Fig. 3a, b). Indeed, Tg mice developed a rapid and lethal cachexia associated with systemic immune disorders: hematopoietic dysregulation (higher absolute number of mature CD11b^+^Ly6G^+^Ly6C^low/int^ and CD11b^+^Ly6G^−^Ly6C^high^ cells), a systemic cytokine storm and massive infiltration of myeloid cells in several organs (Supplementary Fig. 3c–f), associated with a decrease in CD3^+^ T cells and preservation of T regulatory (Treg) lymphocytes (Supplementary Fig. 3g, h). Despite the short life span, these mice allowed us to investigate the relationship between FLIP and chemotherapy in a tumor-free environment. To this end, we added the monocyte-depleting dose of 5-fluorouracil at the beginning of in vitro culture with either wild type (WT) or Tg BM cells and, after four days, determined the absolute numbers of Ly6C^+^ monocytes. Cells with a higher intensity of GFP expression (i.e., cells with higher expression of v-FLIP) survived and were recovered following chemotherapy, compared to those that displayed a lower intensity in GFP (Fig. 2a). Furthermore, v-FLIP-BM-MDSCs retained some suppressive ability after the chemotherapy treatment (Fig. 2b). We also observed that freshly isolated, v-FLIP-expressing-Ly6C^+^ cells displayed an intrinsically higher suppressive ability compared to the WT monocytes on a per cell basis while v-FLIP-expressing-Ly6G^+^ cells did not show any statistically relevant enhancement in their immunosuppressive function (*p* = 0.235) (Fig. 2c). The difference between v-FLIP-expressing Ly6C^+^ and Ly6G^+^ cells was also demonstrated in vivo. MCA205 tumor-bearing mice were treated with anti-PD^−^1 mAb and, later, intravenously transferred with either fresh Ly6C^+^ or Ly6G^+^ cells isolated from the BM of either Tg or WT mice. Only the adoptive cell transfer of v-FLIP-expressing Ly6C^+^ cells was able to abrogate the checkpoint inhibitor efficacy. On the contrary, the transfer of either v-FLIP-expressing- or wild type Ly6G^+^ cells did not result in a statistically significant change (*p* = 0.667 and *p* = 0.687, respectively) (Fig. 2d and Supplementary Fig. 4a). The suppressive ability of the fresh v-FLIP Tg monocytes was further confirmed in an ACT experimental setting where the infusion of v-FLIP-expressing Ly6C^+^ cells completely abrogated the therapeutic efficacy of OVA-specific T cells in controlling EG7-OVA tumor progression (Fig. 2e and Supplementary Fig. 4b). These data indicate that unrestrained expression of v-FLIP, similarly to c-FLIP, is able to confer chemotherapy resistance in Ly6C^+^ cells and reprogram steady-state monocytes to present enhanced immunosuppressive activity.

### Human c-FLIP-engineered monocytes induce in vivo tolerance

**Fig. 3:**
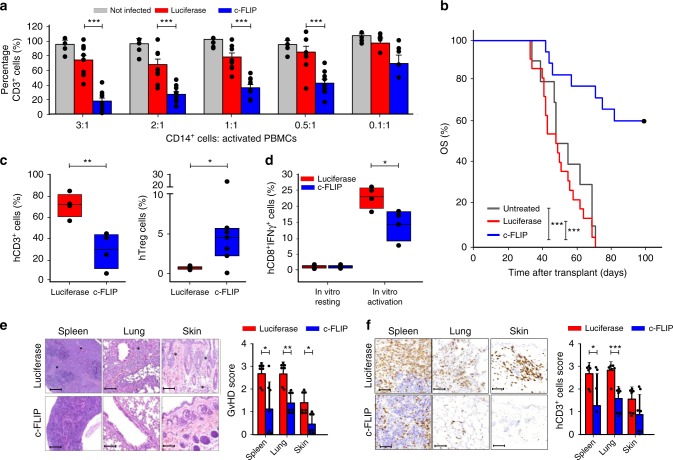
c-FLIP-engineered monocytes transfer controls GvHD progression. **a** Immunosuppressive functions of CD14^+^ monocytes freshly isolated from buffy coats of healthy donors and infected with either luciferase- (as control) or c-FLIP-expressing lentivirus vector was measured by enumeration of absolute number of human CD3^+^ T cells collected after co-culture with engineered monocytes. **b** Kaplan–Meier survival analysis of mice developing xenogeneic GvHD and treated with adoptive cell therapy based on the administration of human engineered, c-FLIP-expressing monocytes: untreated mice, *n* = 10; luciferase-treated mice, *n* = 22 and c-FLIP-treated mice, *n* = 18. **c** Frequency of circulating human CD3^+^ T lymphocytes and Treg (CD3^+^CD4^+^CD25^+^FoxP3^+^ cells) in mice with GvHD treated with the administration of either luciferase- or c-FLIP-expressing monocytes. **d** Percentage of IFN-γ-producing human CD8^+^ T cells isolated from the spleen of mice with GvHD treated with the administration of either luciferase- or c-FLIP-expressing monocytes and stimulated in vitro with polyclonal activators (PMA/IONO). **e** Representative histological analysis of tissues (spleen, lung, and skin) of mice developing GvHD and treated with human-engineered monocytes. Analysis was performed at day 70 after PBMC transplantation. **f** Human CD3^+^ T cell infiltration in tissues (spleen, lung, and skin) of mice developing GvHD and treated with engineered monocytes. Analysis was performed at day 70 after PBMC transplantation. Data are presented either as mean ± s.e.m of five independent experiments (**a**) or as mean ± s.e.m of seven independent samples (**c**–**f**) or as cumulative data of three independent experiments (**b**). *P < 0.05, **P < 0.01; ***P < 0.001; n.s., not significant, by Mann–Whitney test (**a**, **c**–**f**) and by log-rank test (**b**)

We next asked whether human mature monocytes could be reprogrammed by c-FLIP expression to become immunosuppressive. Starting from healthy donor (HD) buffy coats, we purified and infected CD14^+^ monocytes with either c-FLIP- or luciferase-encoding lentiviruses. Also in differentiated CD14^+^ monocytes, c-FLIP expression increased suppressive activity on stimulated T cells (Fig. 3a and Supplementary Fig. 5a). The acquisition of this immune regulatory property did not correlate with a better survival of c-FLIP-expressing monocytes compared to controls, since the percentage of viable (7-AAD^−^Annexin-V^−^) CD14^+^ cells after in vitro culture, either alone or in the presence of activated T lymphocytes, was comparable during the culture time (Supplementary Fig. 5b), indicating that the immune regulatory activity can be separated from the anti-apoptotic properties of c-FLIP, at least in the case of mature monocytes. Moreover, the infection of CD14^+^ cells with lentiviral vectors expressing different anti-apoptotic genes (Bcl-2 or Bcl-xL)^29,30^ did not induce any functional ability to inhibit T cell proliferation (Supplementary Fig. 5c, d). Therefore, FLIP is unique in driving the acquisition of immunosuppressive program in monocytes, which is not directly linked to longer survival.

Given the strong immune regulatory activity endorsed by FLIP expression, we assessed the ability of c-FLIP-infected CD14^+^ monocytes to control the graft vs host disease (GvHD) progression in a setting of xenogeneic transplantation. The infusion of c-FLIP-transduced CD14^+^ cells, controlled established GvHD, resulting in significantly improved long-term survival compared to both untreated mice and mice that received luciferase-expressing CD14^+^ cells (Fig. 3b). The infusion of c-FLIP-expressing monocytes also caused a contraction in the number of circulating human CD3^+^ T cells, provoked the expansion of Tregs and the impairment in IFN-γ production by stimulated CD8^+^ T cells (Fig. 3c, d). The therapeutic impact of the monocyte-induced immune modulation was confirmed by histology and immunohistochemistry (IHC) analyses (Fig. 3e, f and Supplementary Fig. 6a, b). Human CD14^+^ cell infiltration was not detected in any of the analyzed tissues, excluding the in vivo persistence of c-FLIP-infected cells after their infusion (Supplementary Fig. 6c). Importantly from a clinically relevant translational viewpoint, frozen c-FLIP-expressing monocytes retained their immune modulating activity after thawing and injection in mice; in fact, established GvHD progression was better controlled by the engineered monocytes when compared to thawed human Tregs (Supplementary Fig. 6d–f).

### FLIP activates a broad inflammatory pathway in myeloid cells

**Fig. 4:**
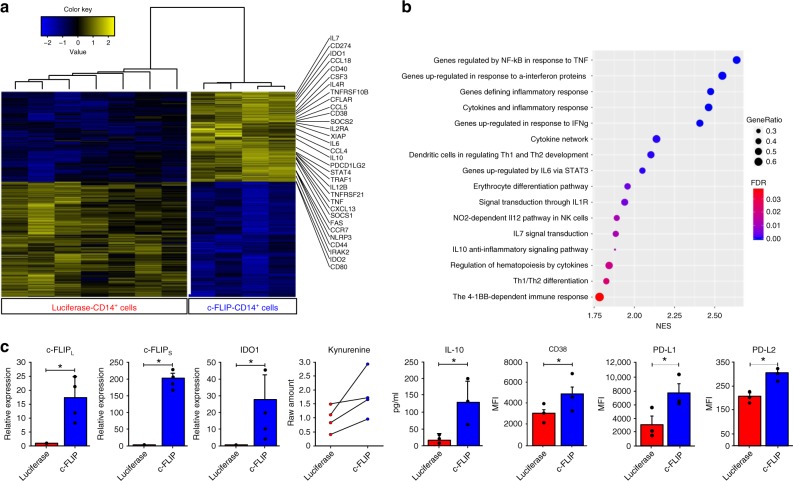
Infected c-FLIP expressing CD14^+^ cells activate MDSC-associated molecular programs. **a** Supervised clustering of c-FLIP arrays using 1,552 differentially expressed genes (FDR <0.01 and absolute fold change >2). **b** Gene set enrichment analysis (GSEA) for association between c-FLIP overexpression and Hallmark (*n* = 50) and Biocarta (*n* = 217) gene sets denoting the activation of specific signaling pathways with a statistical confidence of 95%. NES, normalized enrichment score; FDR, false discovery rate *q*-value; GeneRatio, the percentage of gene hits before the peak in the running enrichment score used as an indication of the percentage of genes contributing to the enrichment score. Dotplot was generated using the ggplot function of the ggplot2 R package. **c** Validation of immune markers induced by c-FLIP in monocytes was performed by real-time PCR (c-FLIP_L_, c-FLIP_S_, and IDO1), by high-performance liquid chromatography (HPLC) in culture supernatants (kynurenine production), by ELISA quantification of culture media (IL-10) and flow cytometry (CD38, PD-L1, and PD-L2). Data are presented as mean ± s.e.m. of five independent experiments (**c**). *P < 0.05, **P < 0.01; ***P < 0.001; n.s., not significant, by Mann–Whitney test

To investigate the molecular events triggered by enforced c-FLIP expression, we analyzed the transcriptomes of c-FLIP- and luciferase-infected CD14^+^ cells (GSE101587). The supervised clustering (q.val < 0.05; fold change >2) indicated more than 1100 upregulated genes associated with immune regulation in c-FLIP-infected monocytes compared to control. The list included *SOCS2, FAS, CCR7, CCL5, STAT3, CD38, CD274, PDCD1LG2, IL6, IL10, CFS3, PTGS2, IDO1*, and *IDO2* that are normally activated in MDSCs^27^ (Fig. 4a). By using the gene set enrichment analysis (GSEA), the differentially expressed genes were significantly enriched in categories involved in inflammation, cytokine network and genes up-regulated in response to interferon proteins, as well as associated to the IL-10 pathway and, overall, to NF-κB-related pathway (Fig. 4b); these categories were confirmed also by gene ontology (GO) enrichment analysis (Supplementary Fig. 7a, b). We proved that c-FLIP-transduced CD14^+^ cells did upregulate both c-FLIP isoforms, i.e. c-FLIP_L_ and c-FLIP_S_, as well as IDO1, and secreted higher amounts of IL-10 and metabolites of IDO1 enzymatic activity, i.e. kynurenine, when compared to luciferase-transduced CD14^+^ cells. Furthermore, c-FLIP-expressing monocytes upregulated the surface molecules PD-L2, PD-L1, and CD38, validating gene expression analysis and highlighting a MDSC-like profile (Fig. 4c).

### The enforced expression of FLIP triggers NF-κB pathway

**Fig. 5:**
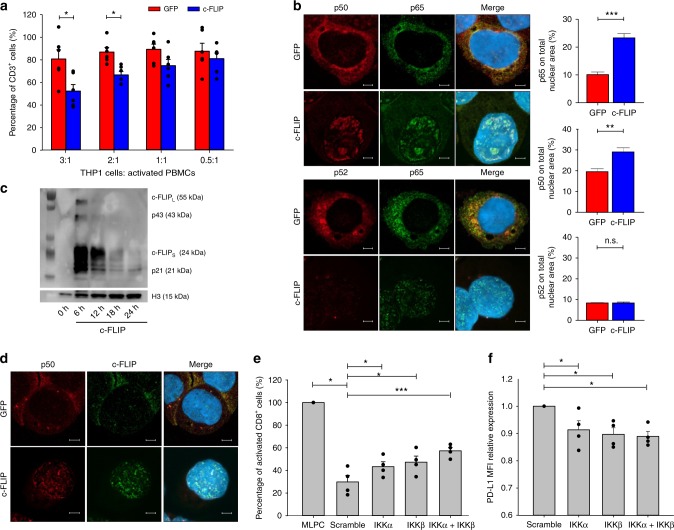
FLIP activates the canonical NF-κB pathway in myeloid cells. **a** Suppressive activity of THP1 cells transfected with c-FLIP RNA was measured by enumeration of an absolute number of human CD3^+^ T cells collected after in vitro co-culture. **b** Immunofluorescence confocal microscopy of p65 (red), p50 and p52 (green) nuclear translocation in transfected THP1 cells. **c** Nuclear c-FLIP protein expression by western blot in THP1 cells transfected with either GFP or c-FLIP RNA at different time points. **d** Immunofluorescence confocal microscopy of p50 (red) and c-FLIP (green) nuclear translocation in transfected THP1 cells. **e** CD11b^+^Ly6C^+^ cells (M-MDSCs) were isolated from the spleen of MCA203 tumor-bearing, wild-type mice by flow sorter and transfected for 18 h with scramble, IKKα, IKKβ, or the combination IKKα plus IKKβ siRNAs. After transfection, cells were washed three times. M-MDSCs were co-incubated with peptide-stimulated CellTrace-labeled OT-I cells. Suppressive activity was measured by enumerating absolute numbers of CD8^+^ T cells collected after in vitro co-culture. **f** PD-L1 expression in transfected M-MDSCs. Data are presented either as mean ± s.e.m of three independent experiments (**a**) or as mean ± s.e.m of four independent experimental transfections (**e**, **f**) where each plot refers to CD11b^+^Ly6C^+^ cells isolated from the pooled spleens of three tumor-bearing mice. Original images × 800 for all panels (**b**, **d**). *P < 0.05, **P < 0.01; ***P < 0.001; n.s., not significant, by Mann–Whitney test (**a**, **b**, **e**, **f**)

Gene expression data suggested a previously unpredicted transcriptional and signaling activity of c-FLIP, possibly linked to the NF-κB pathway. To elucidate the molecular programs under c-FLIP control, we transiently transfected THP1 monocytic cell line with in vitro transcribed (IVT) c-FLIP-encoding RNA. The rational was to induce a sustained and viral-independent production of the target protein; moreover, the IVT-RNA does not activate innate immunity receptors and type I interferon production^31^. Enforced introduction of a synthetic c-FLIP RNA in THP1 cells triggered an immunosuppressive program consistent with the data shown thus far with lentivirus expression vectors (Fig. 5a and Supplementary Fig. 8a). Transfection of c-FLIP RNA significantly promoted the nuclear translocation of the NF-κB subunits p65 and p50, mediators of the canonical NF-κB activation pathway, while no difference in the p52 subunit translocation was detected (Fig. 5b and Supplementary Fig. 8b). The kinetics of protein translocation to nucleus and the activation of a reporter gene in THP1-Blue™ cell line induced by c-FLIP RNA transfection demonstrated a strong signaling (with a top activity at 18 h) even though the c-FLIP cytoplasmic localization peaked at 6 h after transfection (Supplementary Fig. 8c). Unexpectedly, we observed c-FLIP nuclear translocation (Fig. 5c) and, more interestingly, a nuclear co-localization between c-FLIP and p50 (Fig. 5d), which might underlie the gene expression induced by FLIP-enforced expression. Also in freshly isolated, v-FLIP-expressing myeloid cells (Ly6C^+^ and Ly6G^+^ cells) we detected a significant nuclear translocation of p65 and p50 proteins compared to WT cells, especially within the monocytic subset (Supplementary Fig. 8d). These data indicate that v-FLIP signaling modules are shared with c-FLIP and depend on NF-κB activation in monocytes. To test whether NF-κB signaling was required for the immune regulatory program downstream of FLIP, we demonstrated the ability of NF-κB pathway chemical inhibitors (TPCA-1 and 5Z-7-oxozeanol) to abrogate the immunosuppressive activity of v-FLIP-expressing CD11b^+^Ly6C^+^ cells isolated from Tg mice and induce a strong downregulation of membrane PD-L1 (Supplementary Fig. 9a, b). To provide additional, causative evidence that FLIP-driven immunosuppression is mediated by NF-κB, we silenced the kinases IKKα (encoded by *Chuk*) and IKKβ (encoded by *Ikbkb*), alone or in combination (Supplementary Fig. 9c) both indispensable for canonical NF-κB activation. We demonstrated that interference with NF-κB pathway was able to restrain partially the immunosuppressive activity as well as the PD-L1 expression in CD11b^+^Ly6C^+^ cells isolated from either BM of Tg mice (Supplementary Fig. 9d, e) or spleen of tumor-bearing wild-type mice (Fig. 5e, f and Supplementary Fig. 9f). Collectively our data indicate that FLIP activates a signaling pathway that controls the immunosuppressive program in monocytes in part through canonical NF-κB activation, which directly drives PD-L1 expression. We thus confirm and extend the assumption, supported by longstanding experiments on T lymphocytes, about a direct NF-κB activation by c-FLIP, independent from its anti-apoptotic activity^32^.

### FLIP-expressing monocytes in patients with pancreatic cancer

**Fig. 6:**
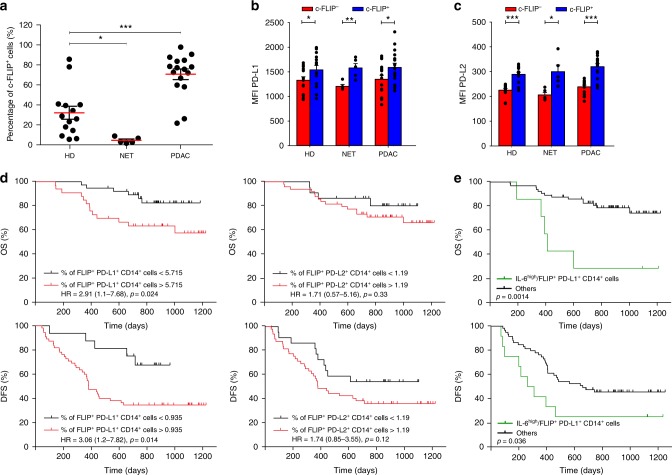
Circulating c-FLIP expressing monocytes increase in PDAC patients. **a** Enumeration of circulating c-FLIP-expressing monocytes (CD14^+^ cells) in healthy donors (HD, *n* = 25) and in neuroendocrine tumor (NET, *n* = 5) and pancreatic ductal adenocarcinoma (PDAC, *n* = 25) patients. **b** PD-L1 expression on HD, NET and PDAC circulating monocytes that express c-FLIP at different levels. **c** PD-L2 expression on HD, NET, and PDAC circulating monocytes that express c-FLIP at different levels. **d** Kaplan–Meier curves for overall survival (OS) and disease-free survival (DFS) in PDAC patients (*n* = 71) by the percentage of FLIP^+^PD-L1^+^CD14^+^ cells and FLIP^+^PD-L2^+^CD14^+^ cells, or **e** high serum levels of IL-6 (IL-6^high^) and FLIP^+^PD-L1^+^CD14^+^ cells. The optimal cutoff thresholds were obtained based on the maximization of the Youden’s statistics (*J* = sensitivity + specificity + 1). *P < 0.05, **P < 0.01; ***P < 0.001; n.s., not significant, by Mann–Whitney test (**a**–**c**). Survival curves were compared by log-rank test (**d**, **e**)

We assessed c-FLIP expression in blood circulating CD14^+^ monocytes, by intracellular staining in combination with surface detection of PD-L1, PD-L2, and CD38 (Supplementary Fig. 10a). We evaluated cohorts of treatment naïve PDAC (*n* = 25) and neuroendocrine tumor (NET, *n* = 5) patients and HDs (patients on oral anticoagulant treatment, *n* = 25), as a control. Compared to HD, there was a significant increase in the percentage of c-FLIP^+^CD14^+^ cells in PDAC but not in NET patients (Fig. 6a), suggesting that c-FLIP might constitute a biomarker for some cancers. Furthermore, heightened surface expression of PD-L1, PD-L2, and CD38 markers was detected in the cell subset identified as c-FLIP^+^CD14^+^ cells, in agreement with our data with genetically-engineered monocytes (Fig. 6b, c and Supplementary Fig. 10b). We evaluated the presence of FLIP-expressing monocytes in frozen PBMCs of a second, independent PDAC patient cohort (*n* = 71) based on the following inclusion criteria: resected pancreatic cancers and treatment naive (Supplementary Tables 1 and 2). Association between the percentage of c-FLIP^+^CD14^+^ cells, overall survival (OS) and disease-free survival (DFS) was explored: a positive, but not statistically significant correlation was observed, probably dependent on the sample size (Supplementary Fig. 10c). However, we demonstrated that PDAC patients with a higher percentage of c-FLIP^+^PD-L1^+^CD14^+^ cells had significantly shorter OS (*p* = 0.024) and DFS (*p* = 0.014) while there was a less significant relationship between FLIP^+^PD-L2^+^CD14^+^ cells, OS and DFS in the same cohort (Fig. 6d). Moreover, a statistically significant correlation was found between the combination of high level of serum IL-6 in conjunction with the frequency of c-FLIP^+^PD-L1^+^CD14^+^ cells and poor OS (*p* = 0.0014) and DFS (*p* = 0.036, Fig. 6e). PD-L1^+^ expression (Supplementary Fig. 10d) and IL-6 serum levels alone^33^ were only predictive for the OS, but not for DFS; instead, PD-L2^+^ expression (Supplementary Fig. 10e) was not predictive for both OS and DFS. Finally, a multivariate analysis indicated that tumor grade G3 and high IL-6/c-FLIP^+^PD-L1^+^CD14^+^ cells were negative independent prognostic factors for both OS and DFS in this patient’s cohort (Supplementary Table 3).

## Discussion

FLIP is an anti-apoptotic regulator that is involved in conferring chemotherapeutic resistance in a wide range of cancers^34^. Our data highlight an inverse correlation between FLIP expression and chemotherapy susceptibility in tolerogenic M-MDSCs both in vivo and in vitro. Tumor-conditioned monocytes, in fact, are so dependent on c-FLIP expression for their survival to be poisoned by treatments reducing the protein expression. We used low doses of different chemotherapeutic drugs, belonging to four classes with diverse mechanisms of action: anti-metabolite agents (5-fluorouracil, gemcitabine), mitotic inhibitors (docetaxel, paclitaxel), alkylating agents (oxaliplatin, cisplatin) and topoisomerase inhibitors (etoposide, irinotecan). All these drugs selectively affected BM-MDSC differentiation, causing a contraction in M-MDSCs (CD11b^+^Ly6C^+^ cells) and loss of immunosuppression via the extrinsic apoptotic pathway, except for fludarabine and carboplatin. Indeed, fludarabine mainly induces apoptosis via a mitochondria-dependent pathway^35^ whereas carboplatin alone has no effect on apoptosis, while it triggers apoptosis via caspase-3 activation in combination with thioridazine^36^. The M-MDSC depletion is mediated by the activation of the caspases cascade belonging to the extrinsic apoptotic pathway and we showed here for the first time, to the best of our knowledge, that c-FLIP is the downstream target of different chemotherapeutics. PMN-MDSCs are likely protected by the exposure to M-MDSC-depleting chemotherapeutics since they do not constitutively require c-FLIP expression for their survival, which instead depends upon Mcl-1^21^.

In addition to influencing cell survival, we clearly defined that c-FLIP directly regulates the tolerogenic properties of monocytes, in part through activation of the canonical NF-κB signaling pathway. Since other anti-apoptotic proteins, such as Bcl-2 and Bcl-xL, are not capable of activating this molecular program, we speculate that FLIP-associated immunosuppression in monocytes is not directly linked to a prolonged survival and thus unveils a previously unrecognized function for this protein. Mouse and human FLIP-expressing monocytes acquired immunosuppressive features able to constrain T cell activation, both in vitro and in vivo. Furthermore, our experiments using a Tg mice, in which the v-FLIP expression was restricted to myeloid cells, demonstrated that FLIP activation during myeloid differentiation induces a severe inflammatory pathology characterized by an unrestrained systemic myeloid cell infiltration that represses B and T cells, resembling the human inflammatory cytokine syndrome (either associated or not with multicentric Castelman disease), a rare heterogeneous disease in part related to Kaposi’s sarcoma herpesvirus infection and characterized by an excess of IL-6 secretion^28^. Interestingly, higher IL-6 serum levels and blood circulating c-FLIP^+^CD14^+^PD-L1^+^ monocytes defined the PDAC patients with worse survival.

Beside the pro-inflammatory gene signature, c-FLIP also affected immune regulatory pathways consisting of more than 1100 upregulated genes, which are enriched in categories involved in NF-κB response to TNF, cytokine network, and genes upregulated by IL-6 via STAT3. This specific gene signature may be linked to a “steered” NF-κB activation induced by the nuclear translocation of FLIP. Moreover, the FLIP-associated signature clustered healthy individuals and cancer patients, reflecting the potential involvement of c-FLIP in cancer-induced inflammation. The enumeration of circulating c-FLIP-expressing monocytes, in association with specific surface markers induced by FLIP, such as PD-L1, PD-L2 and CD38 can thus constitute a useful tool to refine the immunological landscape of cancer patients for a more correct diagnosis and treatment. Obviously, it would be important to exploit the efficacy of these markers in cancer settings other than PDAC.

A careful analysis of the growth curves in tumor-bearing mice treated with anti-PD-1 suggest that PD-L1 expression can be responsible only for a part of the immune evasion mediated by the infusion of v-FLIP-expressing monocytes (Supplementary Fig. 4a). Considering the plethora of immune regulatory circuits activated by FLIP, this was largely expected. Our data suggest that targeting FLIP-associated monocytes in combination with checkpoint inhibitors might increase the therapeutic efficacy of cancer immunotherapy.

By contrast to cancer, where depletion of immunosuppressive myeloid cells is sought, the infusion of engineered, FLIP-expressing monocytes might help treating patients with severe GvHD, with clinical advantages over other immunoregulatory cells, such as Tregs. Several reports suggest the potential effect of MDSC-like cells on GvHD control^37^. In a mouse GvHD model, a single early, post-transplant MDSC infusion transiently suppressed but did not eliminate GvHD^38^. This limited activity likely depends on the infusion of incompletely differentiated myeloid cells, which survive in the host after transfusion and differentiate in vivo into antigen presenting cells (APCs), thus limiting the treatment effectiveness. The M-MDSC subset comprises cycling and partially committed precursors^9^ that can lose their immunosuppressive ability following activation of AIM2 inflammasome^38^. In our experiments, the therapeutic effect of FLIP-expressing human CD14^+^ cells was assessed in a stringent experimental model of established GvHD. In fact, infusions of myeloid cells were started when the disease was clearly detected (circulating hCD3^+^ T cells >5%). FLIP-expressing, human CD14^+^ cells did not survive in vivo for a long time after transfer, limiting their potential to mature into APCs able to sustain T cell activation. We think that all these combined effects allowed reconditioned hCD14^+^ monocytes to control disease progression in the host.

We have uncovered a critical dual role of FLIP in myeloid cell physiology. In mature monocytes, cancer-induced FLIP expression promotes immune suppressive functions, prolongs survival and confers chemotherapy susceptibility; on the other hand, constitutive FLIP activation in myeloid lineage fuels a chronic inflammatory syndrome associated with myeloproliferation and immune suppression. Hence, FLIP emerges as a novel candidate for controlling cancer-associated chronic inflammation and immune dysfunction, as well as a target to generate powerful immune modulators of exaggerated, life-threatening immune responses.

## Methods

### Mice

NOD.Cg-*Prkdc*^*scid*^*Il2rg*^*tm1Sug*^/JicTac (NOG) mice were purchased from Taconic Biosciences (Hudson, NY, USA). C57BL/6J mice were purchased from Charles River Laboratories Inc (Calco, Italy). OT-1 TCR-transgenic mice (C57BL/6-Tg(TcraTcrb)1100Mjb/J) and CD45.1^+^ congenic mice (B6.SJL-PtrcaPepcb/BoyJ) were from Jackson Laboratories (Bar Harbor, ME, USA). Rosa26.vFLIP mice were a gift from Dr. Ethel Cesarman (Weill Cornell Medicine, NY, USA), LysM-CRE mice were a gift from Dr. Patrizia Scapini (University of Verona, Verona, Italy). All mice were maintained under specific pathogen-free conditions in the animal facilities of the University of Verona. Animal experiments were performed according to national (protocol number 12722 approved by the Ministerial Decree Number 14/2012-B of January 18, 2012 and protocol number BR15/08 approved by the Ministerial Decree Number 925/2015-PR of August 28, 2015) and European laws and regulations. All animal experiments were approved by Verona University Ethical Committee (http://www.medicina.univr.it/fol/main?ent=bibliocr&id=85) and conducted according to the guidelines of Federation of European Laboratory Animal Science Associations (FELASA). All animal experiments were in accordance with the Amsterdam Protocol on animal protection and welfare: mice were monitored daily and euthanized when displaying excessive discomfort.

### Human samples

CD34^+^ cells isolated from BM were purchased by AllCell (Alameda, CA, USA). Lin^−^ cells were isolated from fresh BM aspirate samples with normal cytological characteristics, obtained from patients undergoing surgical implants in the Orthopaedic Clinic. The project was approved by the local Ethics Committee (2014/101 on 15/12/2014 [PI]: S.M.) and all patients gave their informed consent. PBMCs isolated from either PDAC and NET patients or Healthy Donors (HD) were collected by informed consent to biobank prior the study (Prot. 52070, Prog. 1885 on 17/11/2010, principal investigator [PI]: A.S.) and the study was approved by the Ethics Committee (Prot. 25978, Prog. 2172 on 29/05/2012, principal investigator [PI]: A.S.).

Indeed, 287 patients admitted at the Unit of General and Pancreatic Surgery of the Azienda Ospedaliera Universitaria Integrata of Verona between 2012 and 2014 with suspected PDAC were assessed for eligibility. Among them, a total of 71 treatment-naïve resectable patients with histologically proven non-metastatic PDAC were included in the study. Inclusion criteria for this study were: histopathological confirmation of PDAC, no prior neo-adjuvant therapy, no evidence of metastatic disease, eligible for surgical resection. Peripheral blood samples were prospectively collected from all patients before surgical resection. Clinic-pathologic features of patients were reported in Supplementary Table 2 and included age, gender, tumor location, tumor size, differentiation status, lymph node involvement and TNM stage, patterns of resection margins, patterns of recurrence. Disease-free survival (DFS) was determined from the time of surgery until local or metastatic PDAC tumor recurrence. Overall survival (OS) was defined as the time of surgery to death. Informed consent was obtained from all subjects. This study was performed in accordance with the ethical standards of the Helsinki Declaration of the World Medical Association. Compliance with REMARK guidelines is reported in Supplementary Table 1. Survival curves were drawn by Kaplan-Meier estimates and compared by log-rank test. The optimal cutoff thresholds of biomarkers were obtained based on the maximization of the Youden’s statistics *J* = sensitivity + specificity + 1 using an R-based software as described in Budczies et al.^39^. Multivariate analyses were conducted by Cox’s proportional hazard regression models using all variables that were significant in univariate analysis (*p* ≤ 0.05). Statistical analyses were performed using GraphPad Prism software program (version 5.00 for MAC OS X, GraphPad Software, San Diego, California USA, www.graphpad.com), IBM SPSS Statistics (version 24.0 Armonk, NY: IBM Corp.), and the statistical language R.

### Cell lines

Chicken ovalbumin (OVA)-transfected EL-4 (EG7) cell line derived from C57BL/6J mice and human embryonic kidney cell line 293 (HEK293, ATCC® CRL-1573™, LGC Standards S.r.l., Milano, Italy), THP-1 cell line (ATCC® TIB-202™, LGC Standards S.r.l., Milano, Italy), THP1-Blue™ NF-κB cell line (InvivoGen, CA, USA), MCA203 cell line, a fibrosarcoma induced by 3-methylcholanthrene (a kind gift by Prof. M.P.Colombo) and MCA205 cell line, a mouse sarcoma (a kind gift by Prof. L. Zitvogel) were grown in DMEM (Invitrogen, Carlsbad, CA, USA) supplemented with 2 mM _L_-glutamine (Euroclone, Milano, Italy), 10 mM HEPES (Euroclone, Milano, Italy), 20 µM β-mercaptoethanol (Sigma-Aldrich, Saint Louis, MO, USA), 150 U/ml streptomycin (Euroclone, Milano, Italy), 200 U/ml penicillin (Euroclone, Milano, Italy) and 10% heat-inactivated fetal bovine serum (FBS; Invitrogen, Carlsbad, CA, USA). MSC2 is an immortalized cell line derived from Balb/c mice^26^ and it was cultured in RPMI 1640 (Life Technologies, Carlsbad, CA, USA) supplemented with 2 mM _L_-glutamine (Euroclone, Milano, Italy), 10 mM HEPES (Euroclone, Milano, Italy), 150 U/ml streptomycin (Euroclone, Milano, Italy), 200 U/ml penicillin (Euroclone, Milano, Italy) and 10% heat-inactivated FBS (Superior, Merck, Darmstadt, Germany). All cell lines were tested to be free from Mycoplasma contamination by PCR screening.

### Cytokines, synthetic peptides, reagents, and chemotherapeutic drugs

Mouse recombinant GM-CSF, mouse recombinant IL-6, human recombinant GM-CSF, and human recombinant G-CSF were purchased from Miltenyi Biotec (Bologna, Italy). K^b^-restricted OVA_257–__264_ peptide (SIINFEKL), was synthesized by JPT (Berlin, Germany). Necrostatin-1, 5Z-7-Oxozeaenol (253863) and TPCA-1 (507475-17-4) were purchased from Sigma-Aldrich (Saint Louis, MO, USA) and pan caspase Inhibitor (Z-VAD-FMK) from R&D Systems (Minneapolis, MN, USA). InVivoMAb anti-mouse PD-1 (RMP1-14) and isotype control (2A3) were purchased from Bioxcell (Lebanon, NH, USA). Chemotherapeutic drugs: 5-fluorouracil (51-21-8); gemcitabine (95058-81-4); docetaxel (114977-28-5); paclitaxel (33069-62-4); oxaliplatin (61825-94-3); cisplatin (15663-27-1), etoposide (33419-42-0), irinotecan (100286-90-6), fludarabine (21679-14-1) and carboplatin (41575-94-4) were purchased by Cayman Chemical (Ann Arbor, MI, USA).

### Mouse proliferation assay

The immunosuppressive activity was evaluated plating in vitro differentiated MDSC or freshly isolated myeloid cells from ROSA26.vFLIP mice in 96 wells plate at a final concentration of 24% of total cells in culture in presence of splenocytes from OT-I transgenic mice, labeled with 1 μM CellTrace (Thermo Fisher Scientific, Waltham, MA, USA) and diluted 1:10 with CD45.1^+^ splenocytes, in the presence of SIINFEKL peptide (1 μg/ml final concentration). After 3 days of co-culture, cells were stained with APC-Cy7 conjugated anti-CD45.2 (clone 104, eBioscience, Thermo Fisher Scientific, Waltham, MA, USA) and PerCP-Cy5.5 conjugated anti-CD8 (clone SK1, eBioscience, Thermo Fisher Scientific, Waltham, MA, USA). CellTrace signal of gated lymphocytes was used to analyze cell proliferation. Samples were acquired with FACS-Canto II (BD, Franklin Lakes, NJ, U.S.A.) using TruCountTM tubes (BD, Franklin Lakes, NJ, USA) to determine the absolute cell number of CD8^+^ cells in the samples. Data were analyzed by FlowJo software (Tree Star, Inc. Ashland, OR, USA).

### Human proliferation assay

PBMCs were isolated from leukocyte-enriched buffy coats from the healthy volunteer (Transfusion Center, University and Hospital Trust of Verona, Verona, Italy) by Ficoll-Hypaque (GE Healthcare, Uppsala, Sweden) gradient centrifugation as described above. The use of PBMC was approved by the ethical Ethic Board of the Azienda Ospedaliera Universitaria Integrata of Verona(AOUI) (protocol numbers: 24114 on 16/05/2017, principal investigator [PI]: V.B.). PBMCs were then counted, frozen at −80 °C and stored in liquid nitrogen. PBMCs were recovered and washed in IMDM medium (Lonza, Visp, Switzerland), supplemented with 10% FBS (Euroclone, Milano, Italy), 100U/ml penicillin/streptomycin (Euroclone, Milano, Italy), β-mercaptoethanol (Sigma-Aldrich, Milan, Italy) and 10 mM HEPES (Euroclone, Milano, Italy). PBMCs were resuspended at a final concentration of 10^7^ cells/ml in PBS and stained with 2,5 μM as final working concentration of CellTrace Violet stock solution (Thermo Fisher Scientific, Waltham, MA, USA), followed by 5 min’ incubation at 37 °C, protected from light. Cells were then washed and resuspended in culture medium. Labeled “target” PBMCs were stimulated with coated 0.6 μg/ml anti-CD3 (clone OKT-3, eBioscience, Thermo Fisher Scientific, Waltham, MA, USA) and 5 μg/ml soluble anti-CD28 (clone CD28.2, eBioscience, Thermo Fisher Scientific, Waltham, MA, USA) for four days and co-cultured with “effectors” CD34^+^ or CD14^+^ cells at 0.1:1, 0.5:1, 1:1, 2:1, 3:1 ratio (effector: target) in 384 flat bottom well plates (BD, Franklin Lakes, NJ, USA). Cell cultures were incubated at 37 °C and 5% CO_2_ in arginine and glutamine–Free-RPMI (Biochrom AG, Berlin, Germany), supplemented with 2 mM L-glutamine (Euroclone, Milano, Italy), 150 μM arginine (Sigma-Aldrich, St. Louis, MO, USA), 10% FBS (Superior, Merck, Darmstadt, Germany), 10 U/ml penicillin and streptomycin (Euroclone, Milano, Italy), and 0.1 mM HEPES (Euroclone, Milano, Italy). At the end of the culture, cells were stained with PE-Cy7 conjugated anti-CD3 (UCHT1, eBioscience, Thermo Fisher Scientific, Waltham, MA, USA), and CellTrace signal of gated lymphocytes was analyzed. TruCountTM tubes (BD, Franklin Lakes, NJ, USA) were used to determine the absolute cell number of CD3^+^ cells in the samples. Data were analyzed by FlowJo software (Tree Star, Inc. Ashland, OR, USA).

### Immunomagnetic human monocytes isolation

Human CD14^+^ monocytes were isolated by immunomagnetic sorting (Miltenyi Biotec, Bologna, Italy) according to the manufacturer’s instructions and their purity was evaluated by flow cytometry using a mouse anti-human mAb.

### Plasmid vectors

Lentiviral transduction vectors pELNS encoding c-FLIP_S_-GFP,c-FLIP_L_-GFP, Bcl-2, and Bcl-xL were a kind gift from Prof. G. Coukos (Department of Oncology, Ludwig Cancer Research Center, University Hospital of Lausanne (CHUV), Lausanne, Switzerland)^17^. Lentiviral transduction vector pELNS encoding luciferase was a kind gift from Dr. D. Melisi (University of Verona). We sub-cloned viral FLIP using plasmids available from Addgene (Cambridge, MA, USA) and then the target sequence into pELNS vector.

### RNA synthesis

The sequences encoding for GFP, c-FLIP_S_, c-FLIP_L_, v-FLIP, eGFP-(2TA)-c-FLIP_S_, eGFP-(2TA)-c-FLIP_L_, eGFP-(2TA)-v-FLIP were subcloned in the pST-T7-hAg-MCS-FI-A30LA70 vectors and IVT mRNA were produced as previously described^31,40^. The 3′ UTR (F-I) of this construct have been shown to enhance stability and translation efficiency, as it has the 100-nucleotide poly(A) tail interrupted by a short linker. Plasmids were linearized with EarI and served as the template for IVT mRNA synthesis using T7 RNA polymerase and a transcription kit (MegaScript, Ambion, Austin, TX, USA). The UTP in the reaction was replaced with 1-methylpseudouridine triphosphate (N1-methylpseudouridine-5′-triphosphate, m1ΨTP) (TriLink). β-S-ARCA(capD1) IVT mRNAs were generated as described elsewhere^41^. Upon IVT mRNA production, single-stranded RNA was enriched by cellulose purification and the absence of double-stranded RNA (dsRNA) was confirmed using the dsRNA-specific mAb J2 (10010200, English and Scientific Consulting)^31^. The quality of the purified dsRNA-free IVT mRNA was assessed by spectrophotometry on a 2100 Bioanalyzer (Agilent), and the mRNA was stored at −80 °C.

### RNA transfection

THP1 RNA-transfection was performed with the following protocol: THP1 were transfected with GFP, c-FLIP_S_-GFP, c-FLIP_L_-GFP, c-FLIP_S_-GFP + c-FLIP_L_-GFP (50 + 50%), v-FLIP-GFP mRNAs using VIROMER RED technology (Lipocalyx, Halle, Germany) according to the supplier instructions: 3 × 10^6^ THP1 cells were seeded in a well of a 6-well plate at a concentration of 2.5 × 10^6^/ml and received 300 μl of a transfection mix composed by 270 μl of mRNA in Viromer Red buffer (11 ng/μl) and 30 μl of Viromer Red solution (diluted 1/25 in Viromer Red Buffer). For mouse monocyte transfection the following protocol was used: after immuno sorting, 1 × 10^6^ Ly6C monocytes were transfected in 1 ml of RPMI 1640 (Life Technologies, Carlsbad, CA, USA) for 4 h with IKKα, IKKβ, or scramble siRNAs using gene silencer technology (GenLantis, San Diego, CA, USA). 4 h post transfection 0.5 ml of media was added. The next day, transfected Ly6C were tested in vitro for their suppressive ability.

### Lentivirus production and infection

Self-inactivating replication of viral particles were produced by calcium phosphate-mediated transfection of packaging cells (HEK293T) with the appropriate amounts of transfer plasmid and HIV-1 lentiviral packaging constructs pDEL, pREV, and VSV-G. Supernatants were harvested 48 h after transfection, 0.22 μm filtered, concentrated by ultracentrifugation (50,000 × *g*; 2,20 h), and titrated by limiting dilution assay in HEK293T.

Human CD34^+^ cells were infected at a MOI (multiplicity of infection) of 2 with 8 µg/ml of polybrene (Millipore, Billerica, MA, USA) and cultured overnight in a 37 °C incubator at 8% CO_2_. Virus-containing media was then removed and replaced with fresh one supplemented with cytokines (G-CSF and GM-CSF at 40 ng/ml each). The cells were then differentiated for 4 days. Human CD14^+^ cells were infected at a MOI of 1 with 8 µg/ml of polybrene (Millipore, Billerica, MA, USA) for 4 h by spin-inoculation (2000 rpm; 37 °C). Transduced cells received fresh media and were incubated overnight in a 37 °C incubator at 5% CO_2_.

### Real-time PCR

Total RNA from both c-FLIP or luciferase- infected monocytes were isolated by TRIzol reagent (Thermo Fisher Scientific, Waltham, MA, USA). The amount and purity of isolated RNA was analyzed by the ND-1000 Spectrophotometer (NanoDrop Technologies). cDNA was prepared using the SuperScript® VILO cDNA Synthesis Kit (Invitrogen, Carlsbad, CA, USA) according to the manufacturer’s instruction. Real Time PCR was run using 2x SYBR Green master mix (ABI). All samples were normalized using GAPDH endogenous control primers. Post-qRT-PCR analysis to quantify relative gene expression was performed by the comparative Ct method (2^−ΔΔCt^).

### Gene expression

For microarrays of genes regulated by c-FLIP overexpression, we prepared *n* = 4 biological replicates of c-FLIP overexpressing cells and *n* = 7 controls. Total RNA was extracted using RNeasy Mini Kit (Qiagen, Hilden, Germany), and contaminant DNA was removed by RNase-Free DNase Set (Qiagen, Hilden, Germany). RNA quality and purity were assessed on the Agilent Bioanalyzer 2100 (Agilent Technologies, Milano, Italy); RNA concentration was determined using the NanoDrop ND-1000 Spectrophotometer (NanoDrop Technologies). As control of effective gene modulation and of the whole procedure, we monitored the expression levels of c-FLIP by qPCR prior to microarray hybridization. Labeling and hybridization were performed according to Affymetrix One Cycle Target Labeling protocol on HG-U133 Plus 2.0 arrays (Affymetrix, Thermo Fisher Scientific, Waltham, MA, USA). Microarray data are available at Gene Expression Omnibus under accession GSE101587 (see also Supplementary Dataset_1).

All data analyses were performed in R (version 3.3.1) using Bioconductor libraries (BioC 3.5) and R statistical packages. Probe level signals were converted to expression values using robust multi-array average procedure RMA^42^ of Bioconductor affy package and batch-corrected using the ComBat function of Bioconductor sva package. To identify genes associated with c-FLIP overexpression in CD14^+^ cells (c-FLIP signature), we compared the expression levels of CD14^+^ cells transfected with c-FLIP with those of control cells using Significance Analysis of Microarray algorithm coded in the same R package^43^. In SAM, we estimated the percentage of false-positive predictions (i.e., false discovery rate, FDR) with 100 permutations and selected as differentially expressed those probe sets with an FDR *q*-value ≤0.01 and an absolute fold change ≥2. This selection resulted in 1690 probe sets corresponding to 486 unique genes upregulated by c-FLIP (c-FLIP signature; GSE101587) and 523 unique downregulated genes. To functionally annotate genes induced by c-FLIP, we consider the Biological Process Gene Ontology (GO) categories and the GO over-representation test^44^ implemented in the Bioconductor cluster Profiler package. GO terms were considered significant at a confidence level of 95% after *p*-value correction using the Benjamini–Hochberg (BH) procedure.

Over-representation analysis was performed using Gene Set Enrichment Analysis and gene sets of the Biocarta and Hallmark collections from the Broad Institute Molecular Signatures Database (http://software.broadinstitute.org/gsea/msigdb). GSEA software (http://www.broadinstitute.org/gsea/index.jsp) was applied on log2 expression data of cells transfected with c-FLIP or with control vector. Gene sets were considered significantly enriched at FDR <5% when using Signal2Noise as metric and 1000 permutations of gene sets.

Average signature expression has been calculated as the standardized average expression of all signature genes in sample subgroups. Signature scores have been obtained summarizing the standardized expression levels of signature genes into a combined score with zero mean^45^. The values shown in graphs are thus a dimensional.

### Elisa assays

ELISA for human IL-10 (eBioscience, Thermo Fisher Scientific, Waltham, MA, USA), ProcartaPlex Mouse Cytokine & Chemokine Panel 1A (36 plex: IFN-γ; IL-12p70; IL-13; IL-1β; IL-2; IL-4; IL-5; IL-6; TNF-α; GM-CSF; IL-18; IL-10; IL-17A; IL-22; IL-23; IL-27; IL-9; GRO-α; IP-10; MCP-1; MCP-3; MIP-1α; MIP-1β; MIP-2; RANTES; Eotaxin; IFNα; IL-15/IL-15R; IL-28; IL-31; IL-1α; IL-3; G-CSF; LIF; ENA-78/CXCL5; M-CSF) (eBioscience, Thermo Fisher Scientific, Waltham, MA, USA) and Bio-Plex Pro Human cytokine standards group II (23-Plex: IL-1β, IL-2, IL-4, IL-5, IL-6, IL-7, IL-8, IL-9, IL-12p70, IL-13, IL-15, IL-17a, eotaxin, IL-1Rα, G-CSF, GM-CSF, IFN-γ, IP10, CCL2, CCL3, CCL4, TNFα, and VEGF) were performed according to manufacturer’s instructions.

### Kynurenine quantification

IDO1 enzymatic activity was measured in vitro in terms of the ability of c-FLIP or luciferase-infected monocytes—incubated for 6 h in a medium supplemented with 100 µM L-Tryptophan (73-22-3; Sigma-Aldrich, Saint Louis, MO, USA)—to metabolize tryptophan to kynurenine, whose concentrations were measured by high performance liquid chromatography (HPLC) in culture supernatants. The detection limit of the assay was 0.05 µM.

### Western blot

Cell lysates were made in RIPA buffer with the addition of protease inhibitor cocktail tablets (Roche, Monza, Italy) and sodium vanadate. Insoluble material was removed by centrifugation. SDS-PAGE was done on 12% denaturing SDS polyacrylamide gel and transferred on PVDF membrane (Millipore, Billerica, MA, USA). Membranes were blocked in Tris-buffered saline plus 0.05% Tween-20 and 5% non-fat milk. We used the following antibodies: anti-mouse caspase-3 (D3E9), caspase-7 (D6H1), caspase-8 (D35G2) and caspase-9 (D2D4), anti-human p65 (D14E12), p50 (D7H5M), p52 (18D10), H3 (D1H2), anti-FLIP (D5J1E) purchased from Cell Signaling Technologies (Danvers, MA, USA), followed by incubation with the secondary goat anti-rabbit IgG antibody, horseradish peroxidase (HRP)-conjugated (Millipore, Billerica, MA, USA). Uncropped images of immunoblots displayed in the figures are in the Supplemntary information section (Supplementary Fig. 11).

### Flow cytometry

Sample tubes were washed in phosphate-buffered saline (PBS) and incubated with 2 µl of FcReceptor Blocking reagent (Miltenyi Biotec, Bologna, Italy) for 10 min at 4 °C to saturate FcR. The following mAbs (1 µl) were then used for cell labeling: anti-mouse CD11b (M170), Ly6C (HK1.4), Ly6G (RB6-8C5), B220 (RA3-6B2), CD3 (145-2C11), CD8 (SK1), CD45.2 (104), CD4 (RM4^−^5), CD25 (BC96), FoxP3 (3G3), Ter-119 (TER-119), CD117 (2B8), IL7RA (A7R34), SCA-1 (D7), CD32 (6C4), CD34 (RAM34); anti-human CD34 (4H11), CD33 (WM53), CD14 (61D3), CD13 (L138), CD15 (MMA), CD16 (3G8), CD11b (ICRF44), CD117 (104D2), CD3 (UCHT1), CD274 (MIH1), CD38 (HIT2), CD273 (MIH18), CD124 (25463), HLA-DR (L243), CD4 (SK3), CD8 (RPA-T8), CD25 (BC96), FoxP3 (259D/C7), IFNγ (B27). All antibodies were purchased from the following companies: BD Biosciences (San Jose, CA, USA), eBiosciences (Thermo Fisher Scientific, Waltham, MA, USA), Biolegend (San Diego, CA, USA) and Cell Signaling Technologies (Danvers, MA, USA). Samples were acquired with a FACSCanto II (BD, Franklin Lakes, NJ, USA) and analyzed with FlowJo software (Treestar Inc.).

FLIP protein expression was evaluated by flow cytometry by indirect amplification on intracellular signal. In details, after surface markers staining, 1 × 10^6^ PBMCs were fixed and permeabilized with Foxp3/Transcription Factor Staining Buffer Set (eBioscience, Thermo Fisher Scientific, Waltham, MA, USA) and 5 µl of purified anti FLIP antibody (D16A8; Cell Signaling Technologies, Danvers, MA, USA) was added for 2 h at 4 °C. Signal was amplified with a secondary anti-rabbit biotin-conjugated antibody (RG-16, 1:1500) Sigma-Aldrich (Saint Louis, MO, USA) and with PerCP-Cy5.5-conjugated Streptavidin (eBioscience, Thermo Fisher Scientific, Waltham, MA, USA) both for 30 min at 4 °C.

### Anti-PD-1 immunotherapy

The effect of anti-PD-1 immunotherapy was investigated in C57BL/6J mice after a s.c. challenge with 8 × 10^5^ MCA205 cells. Tumor-bearing mice with established tumor masses were treated using 4 iterative intraperitoneal administrations every 2 days of anti-PD-1 mAb (clone RMP1-14; complete treatment 1 mg of Ab) or isotype Ab (clone 2A3; complete treatment 1 mg of Ab). After 3 days from the last treatment, we treated some mice by adoptive cell transfer of 1 × 10^6^ cells of: v-FLIP-expressing Ly6C^+^ cells or v-FLIP-expressing Ly6G^+^ cells isolated from tg mice BM. In the control groups we transferred 1 × 10^6^ cells of: Ly6C^+^ cells or Ly6G^+^ cells isolated from WT mice BM. Tumors were measured using digital calipers. Mice were euthanized when tumor area reached 1650 mm^3^.

### Adoptive cell therapy (ACT)

The effects of ACT were investigated in C57BL/6J mice after a s.c. challenge with 5 × 10^5^ EG7-OVA cells. When the tumor area reached ~300 mm^3^, mice received 1 × 10^6^ antigen-activated OVA_257–264_ CTLs by i.v. injection together with 1 × 10^6^ Ly6C^+^ cells isolated from either Tg or WT mice. Recombinant human IL-2 (30,000 IU) was administered twice a day i.p for 3 consecutive days from adoptive transfer^9^. Tumors were measured using digital calipers. Mice were euthanized when tumor area reached 1,000 mm^3^.

### Treg isolation

Thymic (t)Treg were sort-purified from peripheral blood mononuclear cells by Ficoll-Hypaque (GE Healthcare, Uppsala, Sweden) isolated from human apheresis products (Memorial Blood Center, St. Paul, MN) in a two-step procedure whereby CD25^+^ cells (including tTreg) were positively selected using GMP-grade mAb-conjugated magnetic beads (Miltenyi Biotec, Auburn, CA) and then sorted for naïve tTreg (CD4^+^25^++^127^-^45RA^+^). Purified cells were stimulated with GMP-quality artificial antigen presenting cells (aAPC) and cultured for 14 days in high dose IL-2 (300 U/ml) as previously reported^46^. Expanded tTreg were banked (frozen) on day 14. For the experiments described herein, tTreg were thawed and re-stimulated with GMP-compliant anti-CD3/28 beads (Thermo-Fisher Scientific, Waltham, MA) for 7–10 days. tTreg purity and in vitro suppressive function were assessed at the end of the culture. The tTreg are simply indicated as Treg in the text.

### Xenogenic model of GvHD

NOG mice, were irradiated (120 rad) and intravenously (i.v.) injected with 1 × 10^6^ human peripheral blood mononuclear cells. After 21 days, when human CD3^+^ cells where ≥ 5% of total cells, mice were treated by intravenous injection of c-FLIP-, luciferase-infected monocytes or tTreg. The treatment was repeated 4 times on day 21, 28, 42, and 49. Mice were monitored for immunological, clinical and histological scores.

### Confocal microscopy

For immunofluorescence, cyto-spin slides (Shandon™ Single Cytoslides™, Thermo Fisher Scientific, Waltham, MA, USA) were incubated with the following primary antibodies: NF-kB p65 Mouse mAb (#6956, Cell Signaling, Danvers, MA, USA), NF-kB2 p100/p52 Rabbit mAb (#3017, Cell Signaling, Danvers, MA, USA), polyclonal rabbit anti-NF-κB p105/P50 picoband antibody (PB9149, Boster Biological Technology, CA, USA), anti-NFkB p105/p50 antibody (ab32360, Abcam), and monoclonal mouse anti-FLIP antibody (MAB8430, R&D System). Alexa Fluor® 568 goat Anti-Mouse IgG (A11004, Invitrogen, Life Technologies, Carlsbad, CA, US), Alexa Fluor® 488 goat Anti-Mouse IgG (A11029, Invitrogen, Life Technologies, Carlsbad, CA, US), Alexa Fluor® 647 goat Anti-Rabbit IgG (A21244, Invitrogen, Life Technologies, Carlsbad, CA, US), Alexa Fluor® 488 goat Anti-Rabbit IgG (A11008, Invitrogen, Life Technologies, Carlsbad, CA, US), and Alexa Fluor® 546 goat Anti-Rabbit IgG (A11010, Invitrogen, Life Technologies, Carlsbad, CA, US) were used as secondary antibodies. Nuclei were stained with DAPI. Images were acquired with a Zeiss LSM 800 ZEN confocal microscope. The slides were examined in double-blind and digital images of representative areas were taken. The evaluation of NF-kB p65-p50-p52 nuclear positivity was performed on confocal images of 10 THP1 cells and 26–38 Ly6C^+^ or Ly6G^+^ cells per group with Adobe Photoshop by selecting the total nuclear Area with the Lasso Tool, quantifying red or green staining with the Magic Wand Tool and reporting the number of pixels indicated in the histogram window as percentage of the total nuclear area.

### Histology and immunohistochemistry

Tissues were fixed in 10% neutral buffered formalin and embedded into paraffin or fixed in 4% PFA and frozen in a cryo-embedding medium (OCT, BioOptica, MI, Italy); after fixation, bones were decalcified with 10% EDTA (pH 7.4) for 3 weeks and embedded into paraffin; 5 µm thick sections were stained with Hematoxylin and Eosin (BioOptica, MI, Italy) for histological examination. Pathological score was independently evaluated by two pathologists in double-blind using the standard guideline previously published^47^. For immunohistochemistry, mouse anti-human CD3 (LN10, Leica, Wetzlar, Germany), rabbit anti-human CD14 (Sigma-Aldrich,Saint Louis, MO, USA) rat anti-CD11b (550282, BD Pharmingen, San Jose, CA, USA) and rat anti-mouse Foxp3 (14-5773-82, E-bioscience, Thermo Fisher Scientific, Waltham, MA, USA) antibodies were used on cryostat sections while rabbit policlonal anti-CD3 (ab828, Abcam, Cambridge, UK) and rat anti-mouse B220 (550286, BD Pharmingen, San Jose, CA, USA) antibodies were used on paraffin sections. After incubation with the appropriate secondary antibodies, immunostainings were developed with Vulcan Fast Red (Biocare, MI, Italy) alkaline phosphatase or streptavidin peroxidase (Thermo Scientific—Lab Vision Corp.)—DAB Chromogen (Dako, Santa Clara, CA, USA) methods, respectively. After chromogen incubation, slides were counterstained in Hematoxylin (BioOptica, MI, Italy) and images were acquired by Leica DMRD optical microscope (Leica, Wetzlar, Germany).

### Statistical analysis

All data are presented as mean ± standard error of the mean. Statistical analysis was carried out using SigmaPlot (Systat Software). For statistical comparison of two groups, non-parametric Mann-Whitney Wilcoxon test was used. A value of *P* *<* 0.05 was considered significant.

## Electronic supplementary material

Supplementary Information

Supplementary Data 1

## Data Availability

Microarray data are available at Gene Expression Omnibus under accession GSE101587. The authors declare that all the other data supporting the findings of this study are available within the article and its supplementary information files and from the corresponding authors upon reasonable request.
