# Supplementary material for: Induction of immunosuppressive functions and NF-κB by FLIP in monocytes
Source: Nat Commun. 2018 Dec 5;9:5193. doi: 10.1038/s41467-018-07654-4 (PMC6281604; doi:10.1038/s41467-018-07654-4)
Supplement: Supplementary file 1 — Supplementary Information [file 41467_2018_7654_MOESM1_ESM.pdf]

SUPPLEMENTARY INFORMATION.

Induction of immunosuppressive functions and NF-κB by FLIP in monocytes

Fiore et al.

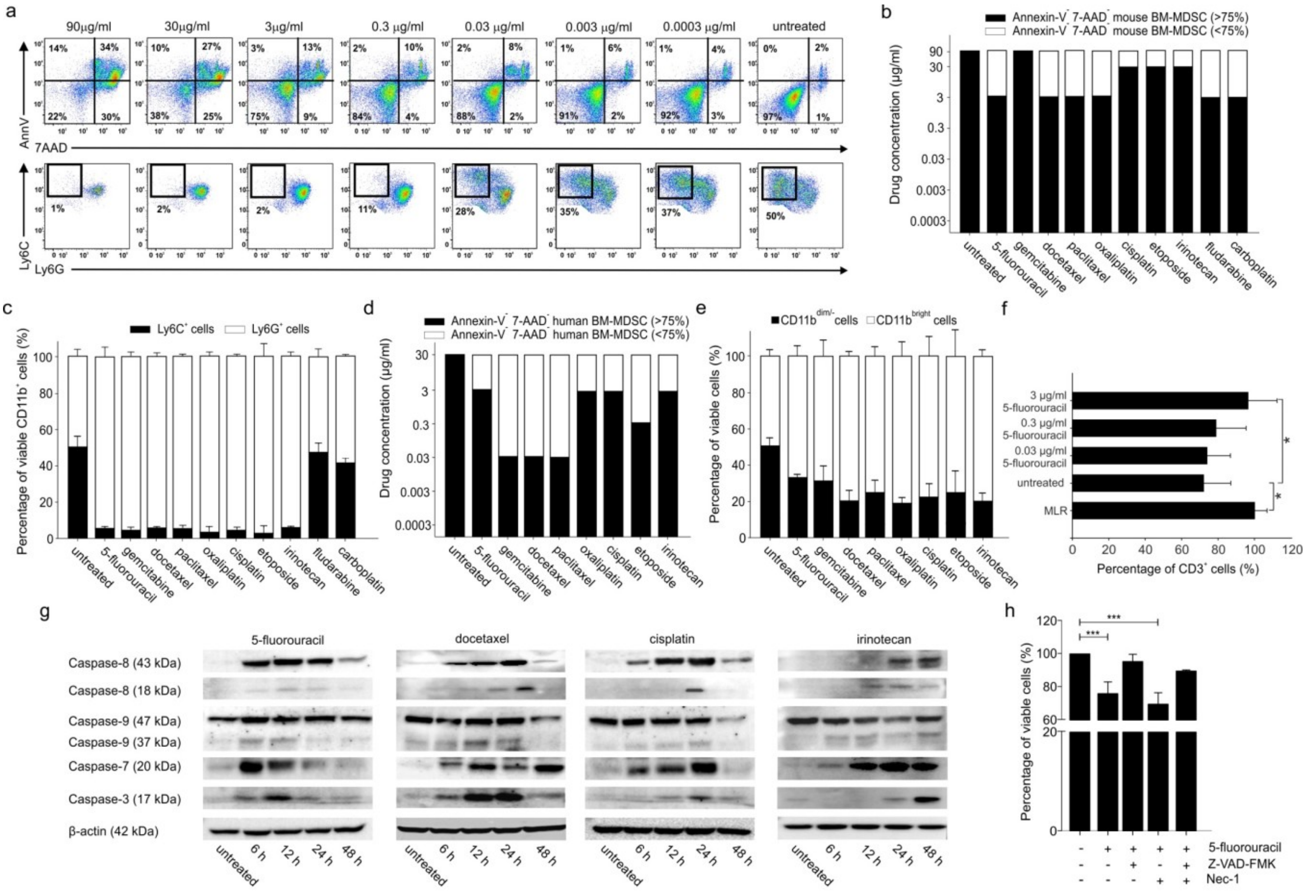

Supplementary Figure 1. Drugs with diverse mechanisms of action activate the extrinsic apoptotic pathway in human and mouse BM-M-MDSCs. a) Representative flow cytometry plots of mouse BM-MDSC viability and phenotype after treatment with different doses of 5-fluorouracil. b) Identification of the highest dose of chemotherapeutics able to preserve the overall mouse BM-MDSC viability (≥ 75% of AnnV$^-$/7AAD$^-$ cells). c) Distribution of M- and PMN-BM-MDSC subsets after exposure to the highest concentration of chemotherapy able to maintain the percentage of viable cells ≥ 75%. d) Identification of the highest dose of chemotherapy able to preserve the human BM-MDSC viability (≥ 75% of AnnV$^-$/7AAD$^-$ cells). e) Distribution of human BM-MDSC subsets, identified as CD11b$^{dim/-}$ cells and CD11b^{bright} cells after exposure to the highest chemotherapy concentration able to maintain the percentage of viable cells ≥ 75%. f) Immunosuppressive activities of human BM-MDSCs treated or not with different doses of 5-fluorouracil. Suppressive activity was measured using CellTrace-labeled PBMCs cultured or not with BM-MDSCs in the presence of anti-CD3 and anti-CD28 mAbs for four days. CellTrace dilution in human CD3$^+$ T cells was evaluated by flow cytometry. Data are presented as mean ± s.e.m. of four independent experiments. g) Caspase activation kinetics in MSC2 cell line after exposure to four chemotherapeutic agents. h) Analysis of the MSC2 cell viability after treatment with a M-BM-MDSC-affecting dose of 5-fluorouracil in the presence of either Z-VAD-FMK (a pan-caspases inhibitor) or necrostatin-1 (Nec-1, a necroptosis inhibitor). Summary data of three independent experiments are presented as mean ± s.e.m. *P < 0.05, **P < 0.01; ***P < 0.001; n.s., not significant, by Mann–Whitney test.

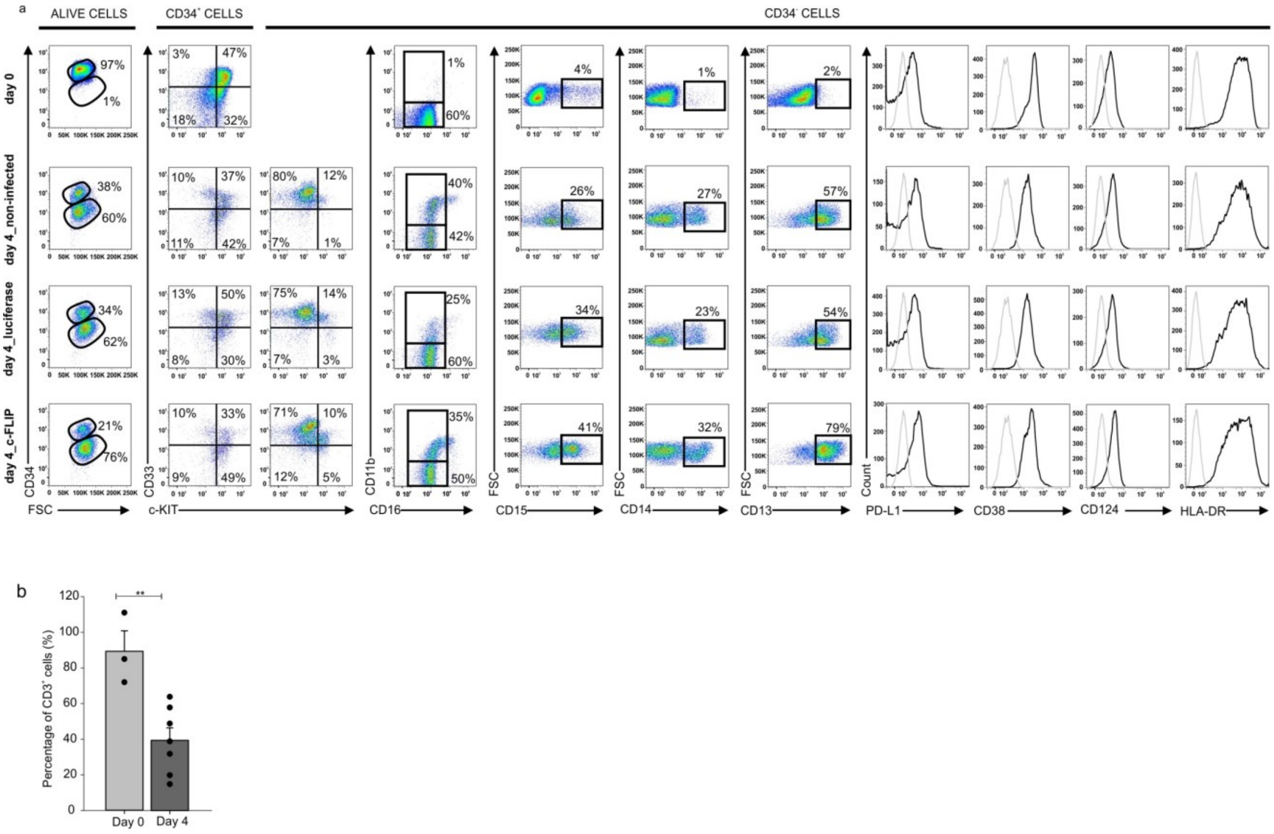

Supplementary Figure 2. Generation of human MDSCs from CD34^{+} stem cells. a) Flow cytometry characterization of *in vitro* generated MDSCs: after four days of *in vitro* culture in the presence of a cytokine cocktail (40 ng/ml of G-CSF plus 40 ng/ml GM-CSF), hCD34^{+} cells differentiate into more mature myeloid cells characterized by the down-regulation of the CD34 marker and the expression of mature myeloid markers such as CD33, CD11b, CD15, CD13, CD14, as well as MDSC-associated markers such as PD-L1, CD38, CD124 and HLA-DR. b) After *in vitro* differentiation, hCD34^{+} cells acquire immunosuppressive activity (dark grey bars) compared to undifferentiated stem cells (light grey bars). Suppressive activity was measured by enumeration of absolute number of CD3^{+} T cells collected after co-culture. Data are presented as mean ± s.e.m. *P < 0.05, **P < 0.01; ***P < 0.001; n.s., not significant, by Mann–Whitney test (b).

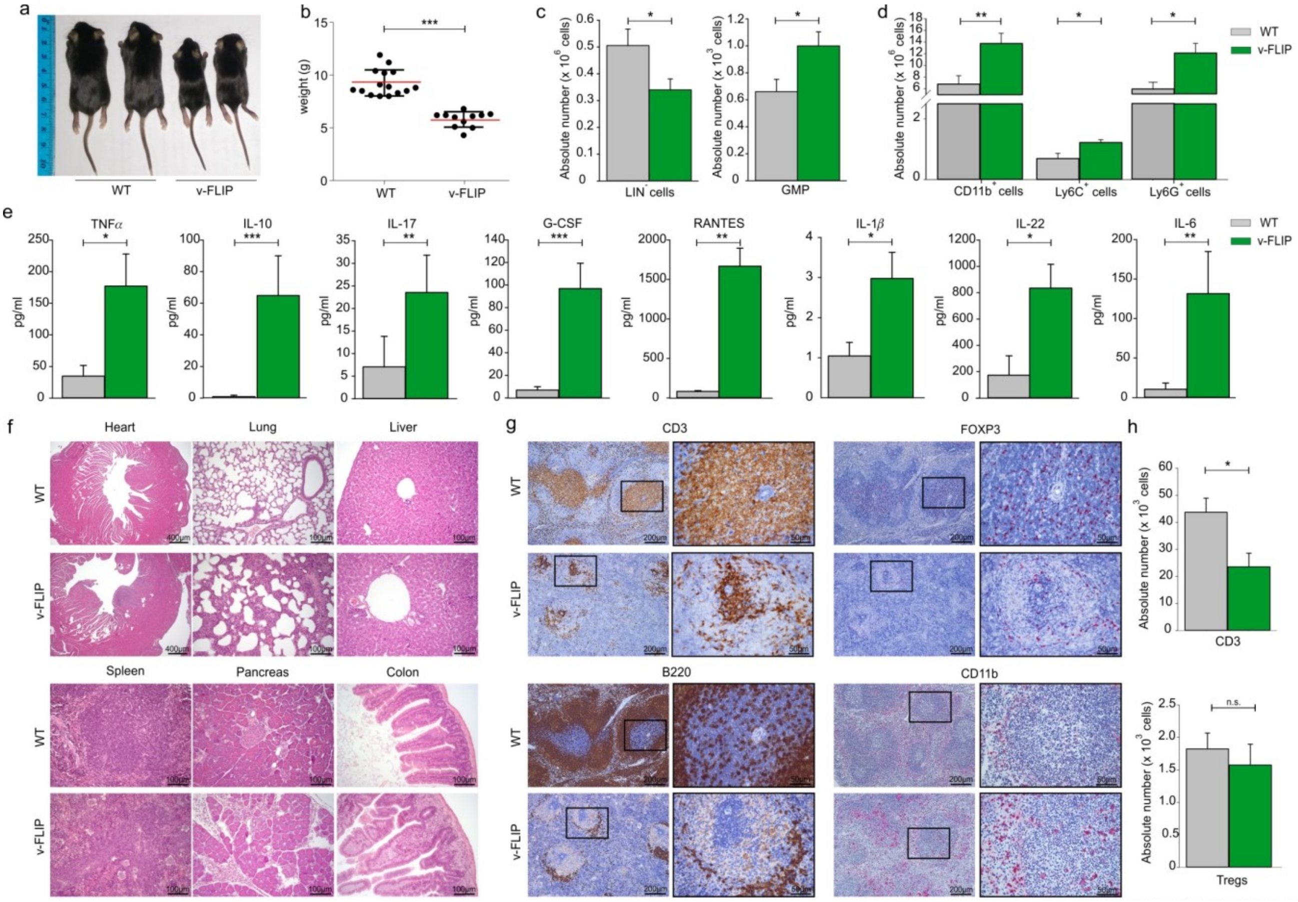

Supplementary Figure 3. The enforced expression of v-FLIP in mouse myeloid precursors favors the development of a myelodysplastic syndrome. a) ROSA26.vFLIPTg; LysM-Cre mice (defined as Tg mice) developed a rapid and lethal cachexia and died at 4th week of age. b) Body weight of WT (n=15) and Tg (n=20) mice of 4th week of age. c) Absolute numbers of myeloid progenitors in WT and Tg mice. d) Absolute numbers of CD11b$^+$, Ly6C$^+$ and Ly6G$^+$ cells in WT (n=8) and Tg (n=8) mice. e) Perturbation of serum cytokines in Tg mice compared to WT mice. Data are representative of 10 Tg and 10 control mice. f) Representative histological analysis of different tissues (heart, lung, liver, spleen, pancreas, colon) of WT and Tg mice demonstrated an extensive myeloid cell accumulation. g) IHC analysis of immune cell distribution in the spleen of Tg mice compared to WT spleen; the panel shows T cells (CD3), B cells (B220), Treg (FoXP3) and myeloid cells (CD11b). h) Absolute numbers of CD3$^+$ and CD3$^+$CD4$^+$CD25$^+$FoXP3$^+$ cells referred as Treg cells by flow cytometry analysis in WT (n=8) and Tg (n=8) mice. Data are presented as mean ± s.e.m. *P < 0.05, **P < 0.01; ***P < 0.001; n.s., not significant, by Mann–Whitney test (b,c,d,e,h).

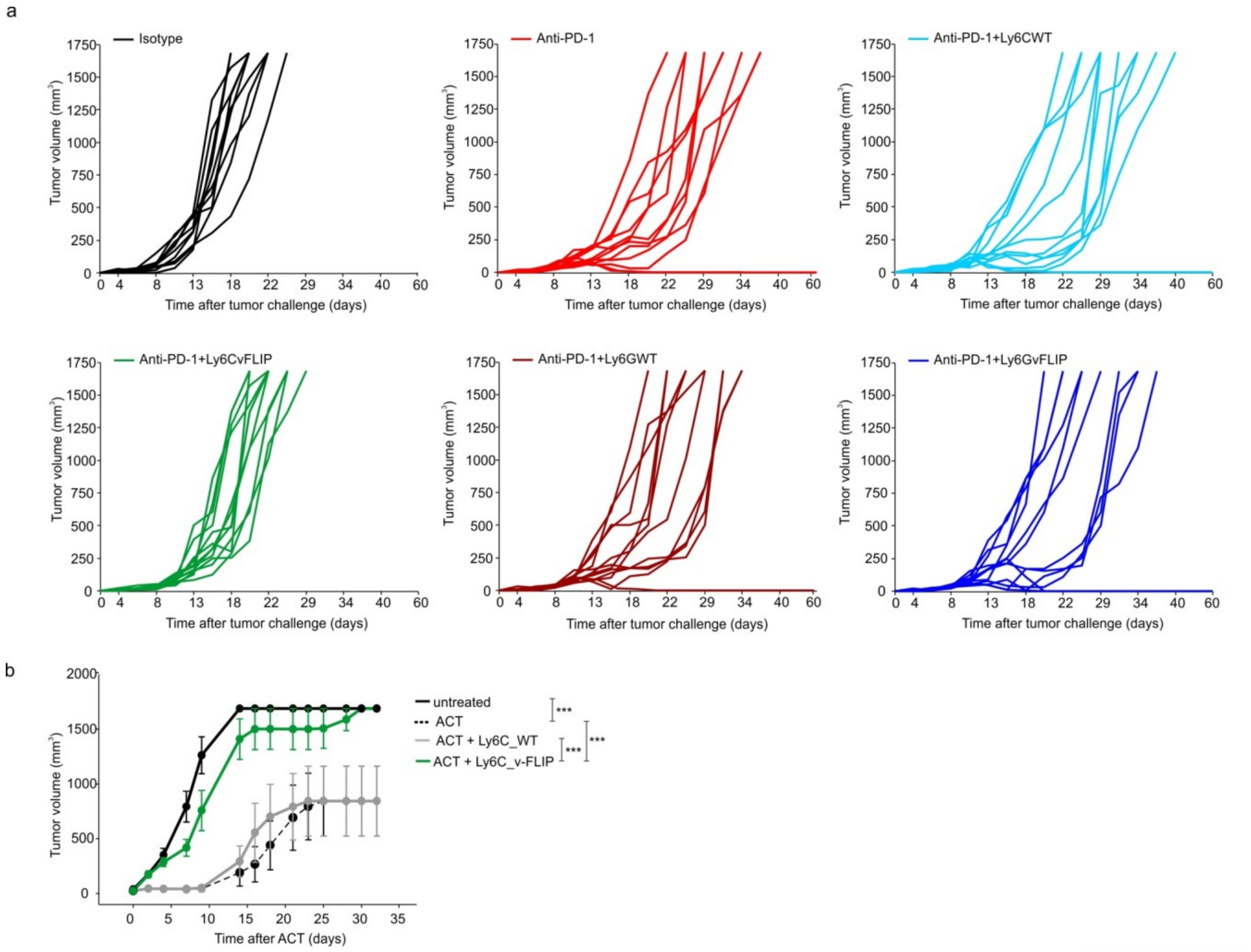

Supplementary Figure 4. Adoptive transfer of v-FLIP expressing Ly6C$^+$ cells abrogates cancer immunotherapy. a) Seven week old C57BL/6J mice were subcutaneously injected with MCA205 tumor cells. Tumor-bearing mice with established tumor masses were treated using 4 iterative, intraperitoneal administration of anti-PD-1 mAb (red lines) or isotype Ab (black lines) every 2 days. Three days after the last treatment, some mice received the adoptive transfer of 1x10^6 cells of either v-FLIP-expressing Ly6C$^+$ cells (green lines) or v-FLIP-expressing Ly6G$^+$ cells (dark blue lines) freshly isolated from the BM of Tg mice. In the control groups, we transferred 1x10^6 cells of either Ly6C$^+$ (light blue lines) or Ly6G$^+$ cells (brown lines) freshly isolated from the BM of WT mice. Mice treated with anti-PD-1 mAb + v-FLIP-Ly6C$^+$ cells (green lines) displayed the same tumor growth of isotype control-treated mice (black lines). Two cumulated experiments are shown (n= 12 mice/group). b) Seven week old C57BL/6J mice were subcutaneously injected with EG7 tumor cells. After 10 days (tumor volume ~9 mm^3) mice were injected intravenously (i.v.) with 10^6 OVA-specific OT-I T lymphocytes (ACT) and, after 2 hours, mice were infused i.v. with 0.5x10^6 fresh CD11b$^+$Ly6C$^+$ monocytes isolated from the BM of either WT or Tg mice. Mice treated with ACT + vFLIP-Ly6C$^+$ cells (green line) displayed the same tumor growth of untreated mice (black line). A representative experiment is shown (n=5 mice/group). *P < 0.05, **P < 0.01; ***P < 0.001; n.s., not significant by one-way analysis of variance (ANOVA).

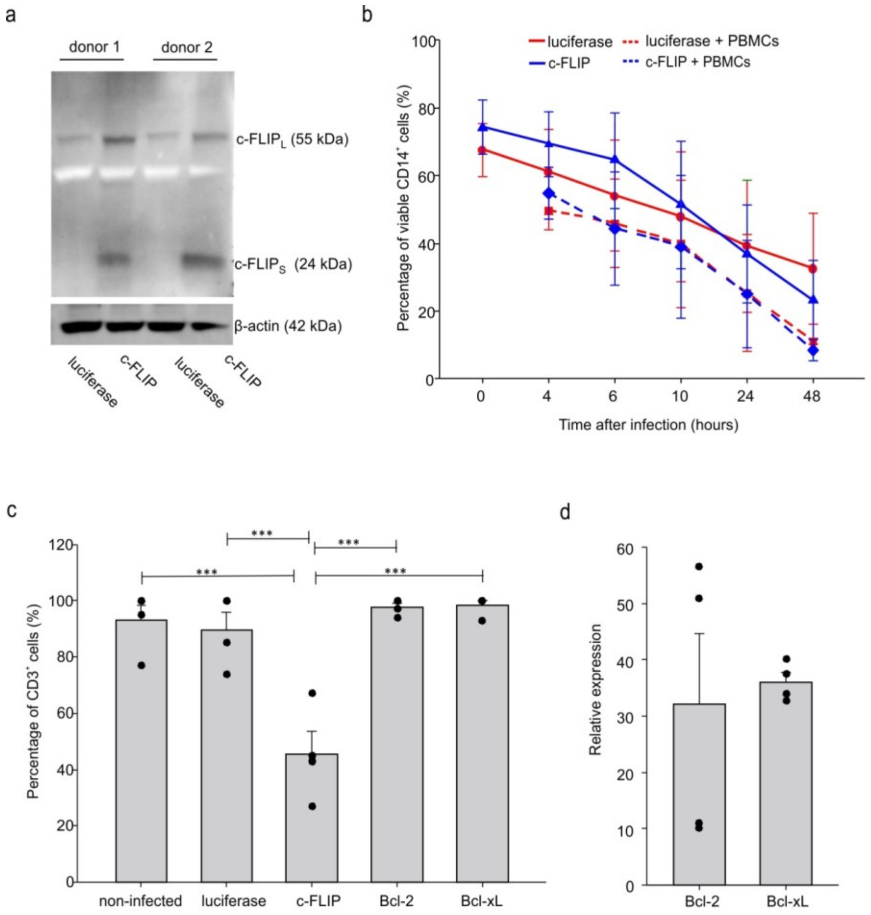

Supplementary Figure 5. Enforced expression of c-FLIP does not prolong human monocyte survival but promotes the acquisition of immunosuppressive properties. a) Western blot analysis of c-FLIP in human monocytes after either luciferase- or c-FLIP-encoding lentivirus infection. Two different monocyte donors are shown. b) Flow cytometry analysis of luciferase- (red line) or c-FLIP- infected (blue line) monocytes viability ($AnnV^-/7\text{-}AAD^-$), from 0 to 48 hours of *in vitro* culture, alone (full lines) or in presence of activated T lymphocytes (dotted lines). c) Immunosuppressive functions of $CD14^+$ monocytes freshly isolated from buffy coats of healthy donors and either not infected or infected with luciferase-, c-FLIP-, Bcl-2- or Bcl-xL-expressing lentivirus vectors. Suppressive activity was measured by enumeration of absolute number of human $CD3^+$ T cells collected after co-culture with the monocytes. Data are presented as mean ± s.e.m of four independent experiments. d) Validation of Bcl-2 or Bcl-xL expression in monocytes was performed by real-time PCR. Data are presented as mean ± s.e.m. of five independent experiments. *P < 0.05, **P < 0.01; ***P < 0.001; n.s., not significant, by Mann–Whitney test (c).

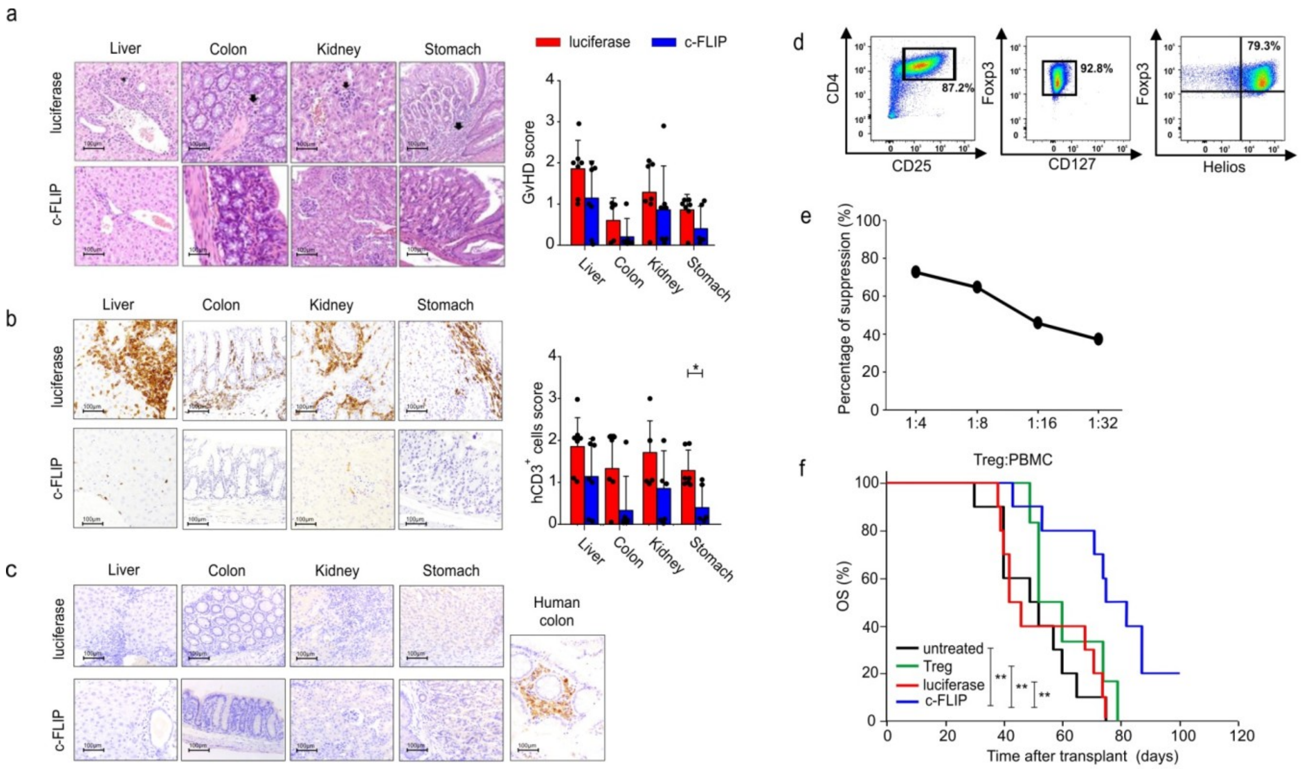

Supplementary Figure 6. Human c-FLIP-expressing monocytes control xenogeneic GvHD. a) Representative histological analysis of tissues (liver, colon, kidney and stomach) of mice developing GvHD and treated with human engineered monocytes. Analysis was performed at day 70 after PBMC transplantation. b) Human $CD3^+$ T cell infiltration in tissues (liver, colon, kidney and stomach) of mice developing GvHD and treated with engineered monocytes. Analysis was performed at day 70 after PBMC transplantation. c) Human $CD14^+$ cell infiltration in tissues (liver, colon, kidney and stomach) of mice developing GvHD and treated with engineered monocytes. Analysis was performed at day 70 after PBMC transplantation. Data are presented as mean ± s.e.m. of 7 mice treated with luciferase-expressing monocytes and 7 mice treated with c-FLIP-expressing monocytes. d) Flow cytometric analysis of human Tregs purified from PBMCs by apheresis. e) Immunosuppressive functions of human Tregs after thawing and re-stimulation with GMP-compliant anti-CD3/28 beads. f) Kaplan-Meier survival analysis of mice developing xenogeneic GvHD and treated with the transfer of human freshly thawed, engineered luciferase- (red line) or c-FLIP-expressing (blue line) monocytes or Tregs (green line). Data show a representative experiment: Untreated mice, n=10; luciferase-treated mice, n=10, c-FLIP-treated mice, n=10 and Treg-treated mice, n=6. *P < 0.05, **P < 0.01; ***P < 0.001; n.s., not significant, by Mann–Whitney test (a,b) and by log-rank test (f).

a

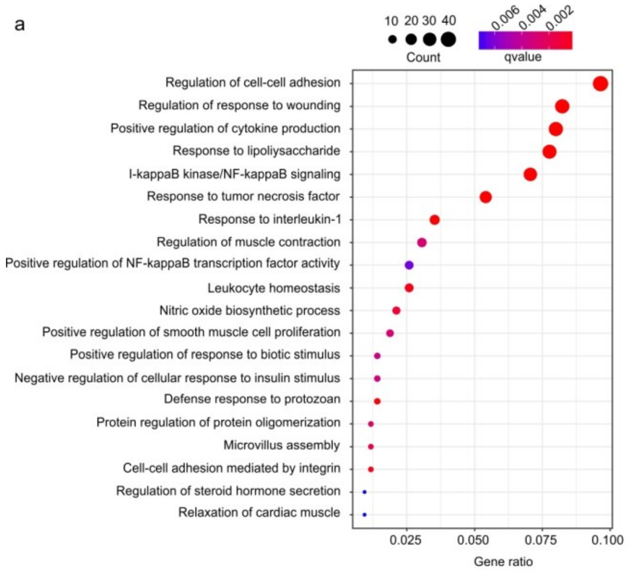

b

Pathway	p-value	FDR
Genes regulated by NF-kB in response to TNF	6.40E-33	3.77E-35
Genes defining inflammatory response	3.96E-14	4.66E-16
Genes up-regulated in response to IFNg	1.18E-13	2.09E-15
Genes up-regulated by IL6 via STAT3	8.09E-10	1.90E-11
Genes up-regulated by STAT5 in response to IL2 stimulation	1.35E-09	3.98E-11
Genes up-regulated during transplant rejection	5.83E-08	2.06E-09
Genes mediating programmed cell death (apoptosis) by activating of caspases	3.06E-05	1.26E-06
Cytokines and inflammatory response	5.02E-05	2.36E-06
Genes up-regulated in response to a-interferon proteins	1.02E-03	5.87E-05
Genes encoding components of the complement system	1.02E-03	5.97E-05
Antigen dependent B cell activation	1.39E-03	9.00E-05
Caspase cascade in apoptosis	2.45E-03	1.73E-04
Genes involved in p53 pathways and networks	2.62E-03	2.16E-04
Genes up-regulated byKRAS activation	2.62E-03	2.16E-04
HIV-I Nef: negative effector of Fas and TNF	3.45E-03	3.05E-04
IL10 anti-inflammatory signaling pathway	6.16E-03	5.80E-04
Th1/Th2 differentiation	7.31E-03	7.31E-04
Cytokine network	0.011	1.12E-03
Genes up-regulated by activation of hedgehog signaling	0.012	1.32E-03
Dendritic cells in regulating Th1 and Th2 development	0.014	1.62E-03
IL-2 receptor β chain in T cell activation	0.016	1.93E-03
Genes encoding components of apical junction complex	0.016	2.07E-03
Genes up-regulated in response to low oxygen levels (hypoxia)	0.019	2.56E-03
CTL mediated immune response against target cells	0.022	3.17E-03
Keratinocyte differentiation	0.025	4.09E-03
Regulation of hematopoiesis by cytokines	0.025	3.96E-03
Selective expression of chemokine receptors duting T-cell polarization	0.025	4.06E-03
Stress induction of HSP regulation	0.029	4.86E-03
Signal transduction through IL1R	0.036	6.62E-03
IL7 signal transduction	0.037	7.02E-03
Genes involved in processing of drugs and other xenobiotics	0.039	7.51E-03

Supplementary Figure 7. Enforced c-FLIP expression in monocytes drives MDSC-associated molecular programs. a) Enrichment of GO BP terms for the 486 unique up-regulated genes in c-FLIP overexpressed samples. Enrichment has been determined using the GO over-representation test implemented in cluster Profiler and the GO Biological Process categories. The p-values have been corrected using BH procedure. b) Enrichment of signaling pathways for up-regulated genes in c-FLIP overexpressed samples. Differentially expressed genes were identified using SAM. Setting q-value<1% resulted in 486 unique genes up-regulated in c-FLIP overexpressed samples (c-FLIP signature). Enrichment was determined using a Fisher's test on all 267 signaling pathways of GSEA analysis (see legend of Figure 4b). The p-values were corrected using BH procedure. FDR q-val, false discovery rate q-value.

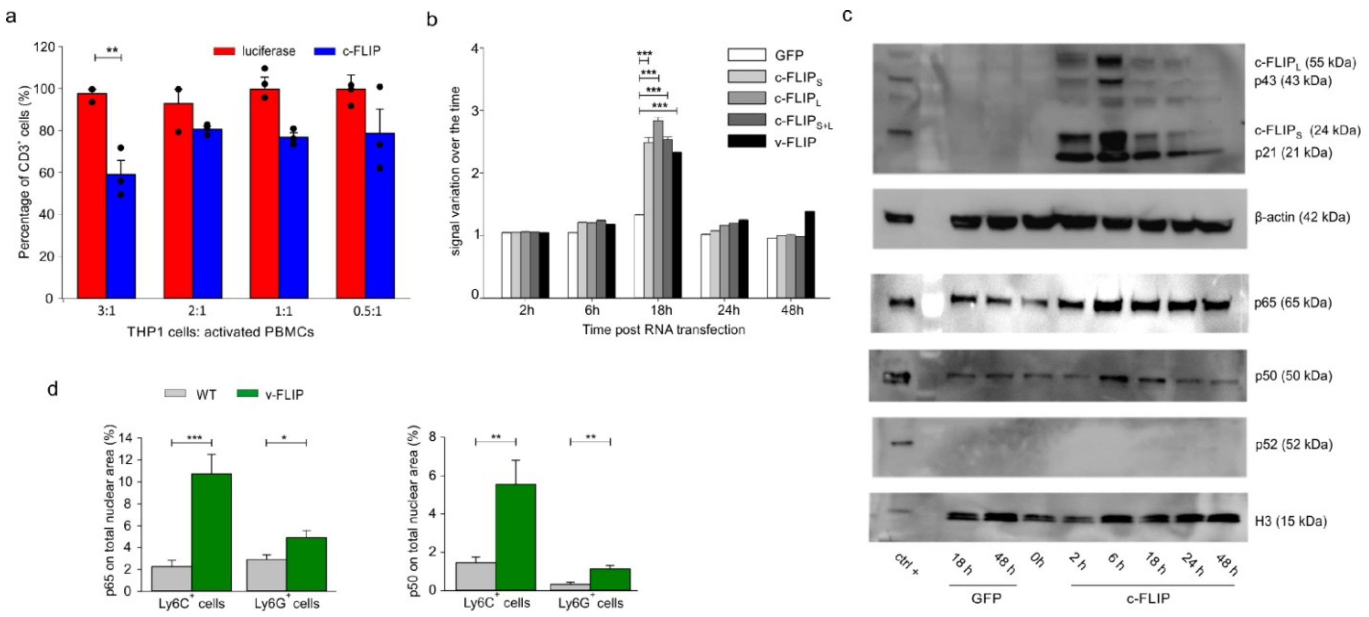

Supplementary Figure 8. FLIP activates the canonical NF-κB pathway in myeloid cells. a) THP1 cells infected with c-FLIP-encoding lentivirus acquired immunosuppressive functions compared to control (THP1 cells infected with luciferase-encoding lentivirus). Suppressive activity was measured by enumeration of absolute number of $CD3^+$ T cells collected after co-culture with engineered THP1 cells. Data are presented as mean ± s.e.m of three independent experiments. b) c-FLIP protein expression validation by western blot in THP1 cells transfected with GFP or c-FLIP RNA at different time points. Protein expression of p65, p50 and p52 was evaluated in the nucleus of THP1-cells transfected with either GFP or c-FLIP RNA at different time points. c) Quantification of NF-κB activation by determining the activity of secreted embryonic alkaline phosphatase (SEAP) in THP1-Blue™ NF-κB cells supernatant after GFP, v-FLIP, or c-FLIP RNA transfection. THP1-Blue™ were derived from the human THP1 monocyte cell line by stable integration of an NF-κB -inducible SEAP reporter construct. THP1-Blue™ NF-κB cells express SEAP reporter gene driven by an IFN-β minimal promoter fused to five copies of the NF-κB consensus transcriptional response element and three copies of the c-Rel binding site. An activation of NF-κB pathway is detectable after 18h from transfection only with FLIP-encoding RNAs. d) Immunofluorescence confocal microscopy of p65 and p50 nuclear translocation in $Ly6C^+$ and $Ly6G^+$ cells purified from the BM of either WT or Tg mice. Data are presented as mean ± s.e.m of four independent experiments. *P < 0.05, **P < 0.01; ***P < 0.001; n.s., not significant, by Mann–Whitney test (a,c,d).

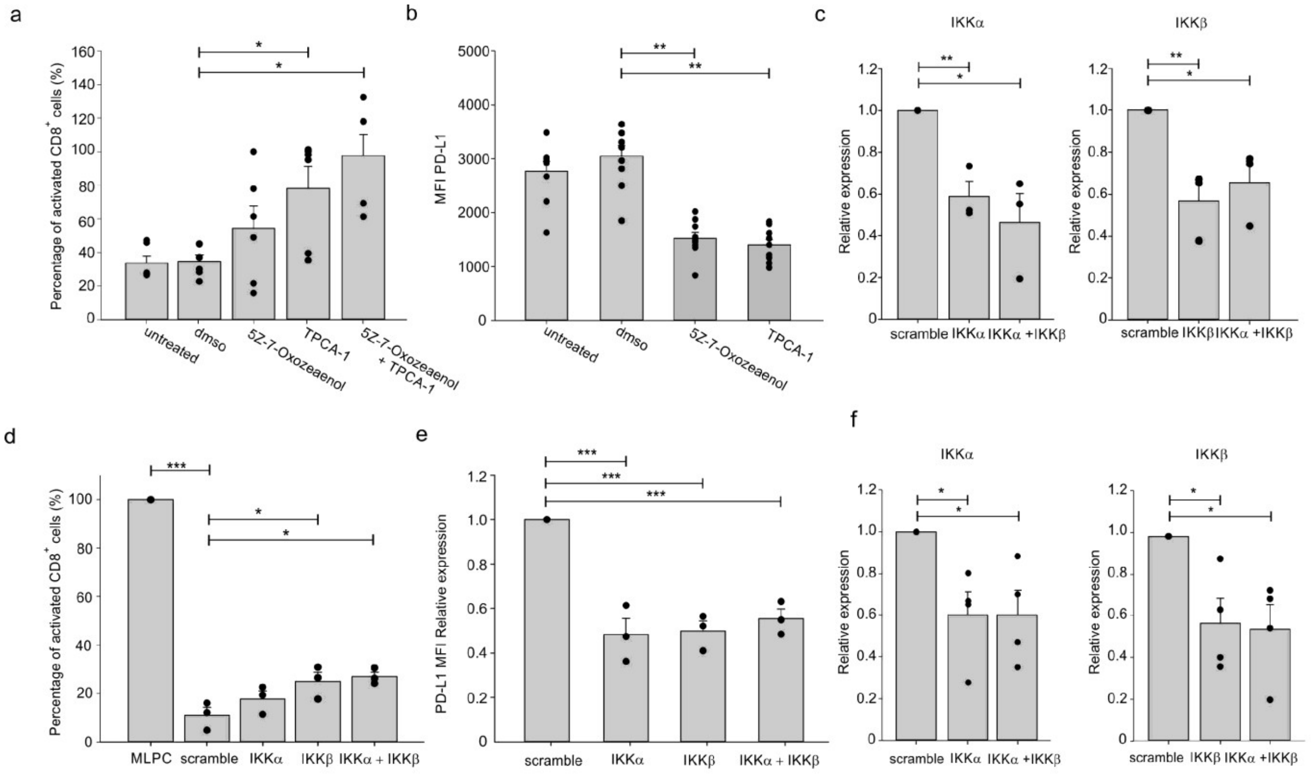

Supplementary Figure 9. Efficacy of NF-κB interference in FLIP-expressing CD11b$^+$Ly6C$^+$ cells. a) v-FLIP-expressing CD11b$^+$Ly6C$^+$ cells were treated with NF-κB inhibitors (5-Z-7-oxozeaenol at 1 µM; TPCA-1 at 5 µM) overnight and incubated with CellTrace-labeled OT-I cells and co-cultured in the presence of SIINFEKL peptide for three days. Suppressive activity was measured by enumeration of absolute number of CD8$^+$ T cells collected after co-culture. b) PD-L1 expression in v-FLIP-expressing CD11b$^+$Ly6C$^+$ cells treated overnight with NF-κB inhibitors. c) v-FLIP-expressing CD11b$^+$Ly6C$^+$ cells purified from the BM of Tg mice were transfected for 18h with scramble, IKKα, IKKβ or the combination IKKα plus IKKβ siRNAs. After transfection, cells were washed three times and the efficacy of target gene down-regulation was evaluated by real-time PCR. d) Following transfection, v-FLIP-expressing CD11b$^+$Ly6C$^+$ cells were incubated with CellTrace-labeled OT-I cells and co-cultured in the presence of SIINFEKL peptide for three days. Suppressive activity was measured by enumerating absolute numbers of CD8$^+$ T cells collected after co-culture. e) PD-L1 expression in transfected v-FLIP-expressing CD11b$^+$Ly6C$^+$ cells. Data are presented as mean ± s.e.m of three independent experiments of transfection in which each plot refers to a pool of CD11b$^+$Ly6C$^+$ cells isolated from BM of (c,d,e). f) CD11b$^+$Ly6C$^+$ cells were isolated from the spleens of MCA203 tumor-bearing wild type mice by flow sorter and transfected for 18h with scramble, IKKα, IKKβ or the combination IKKα plus IKKβ siRNAs. After transfection, cells were washed three times and the efficacy of target gene down-regulation was evaluated by real-time PCR. Data are presented as mean ± s.e.m of four independent experiments of transfections in which each plot refers to a pool of purified CD11b$^+$Ly6C$^+$ cells isolated from three spleen of tumor-bearing mice (f). *$P < 0.05$, **$P < 0.01$; ***$P < 0.001$; n.s., not significant, by Mann–Whitney test.

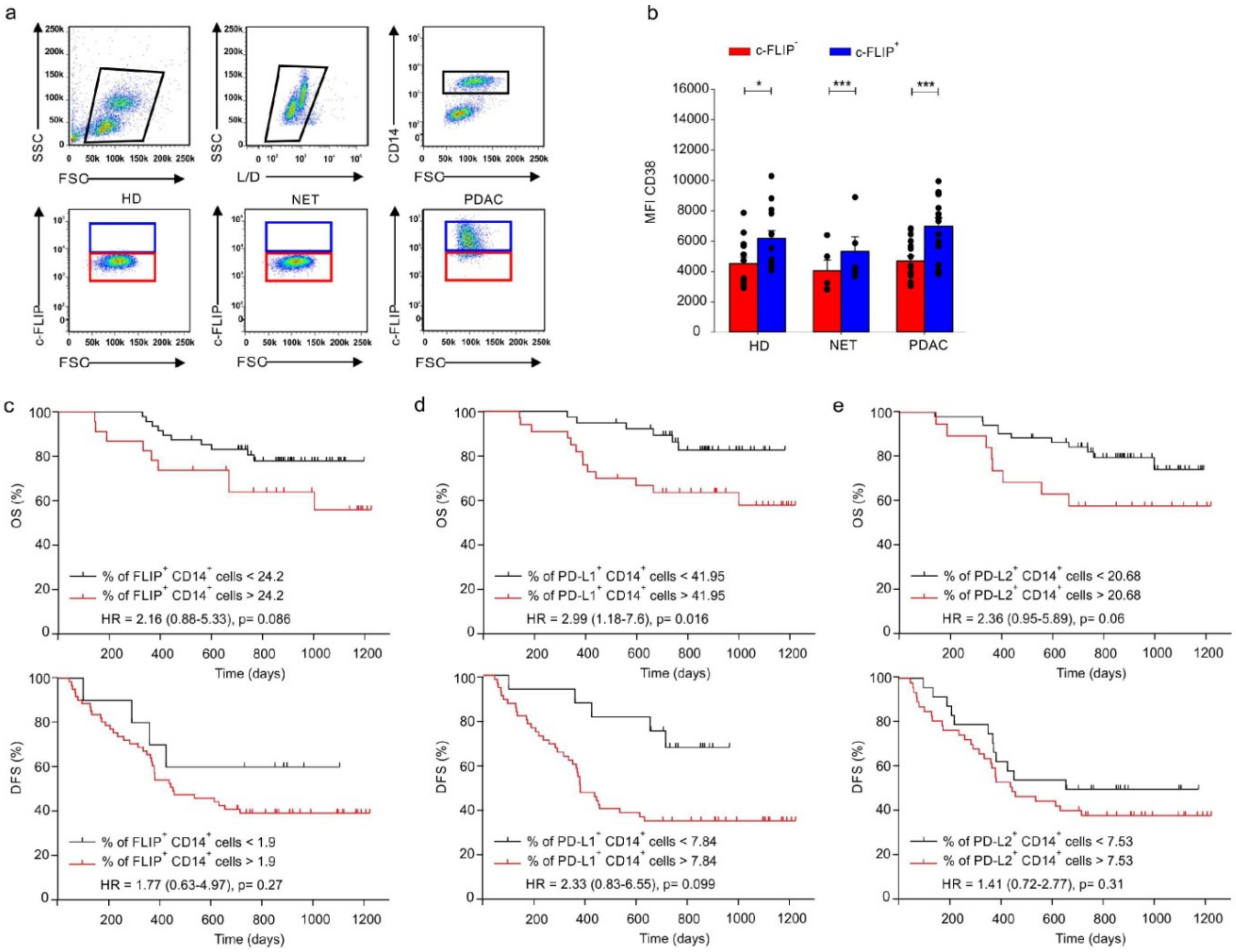

Supplementary Figure 10. Circulating c-FLIP-expressing monocytes increase in PDAC patients. a) Gating strategy to identify c-FLIP expressing cells among blood circulating monocytes ($CD14^+$ cells). Monocytes that express c-FLIP (blue square) and monocytes that do not express c-FLIP (red square) were discriminated by the gating strategy. b) CD38 expression on HD, NET and PDAC circulating monocytes that express c-FLIP at different levels. c) Kaplan-Meier curves for overall survival (OS) and disease free survival (DFS) in PDAC patients (n=71) by the percentage of $FLIP^+CD14^+$, d) $PDL\text{-}1^+CD14^+$, or e) $PDL\text{-}2^+CD14^+$ cells. The optimal cutoff thresholds were obtained based on the maximisation of the Youden's statistics (J=sensitivity+specificity+1). *P < 0.05, **P < 0.01; ***P < 0.001; n.s., not significant, by Mann–Whitney test (b). Survival curves were compared by log rank test (c, d, e).

Figure 5 C

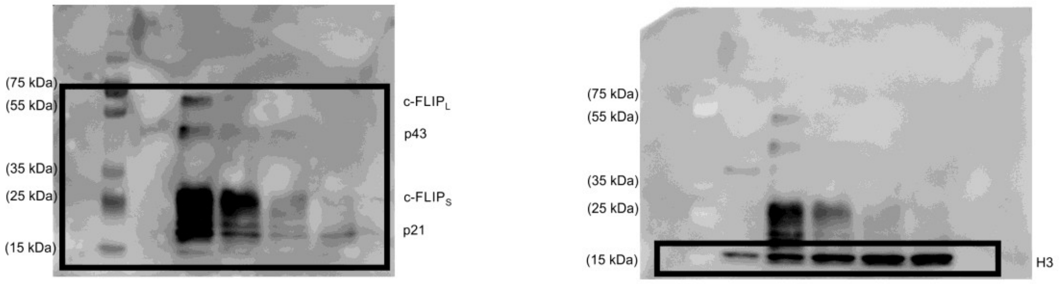

Supplementary Figure 8 C

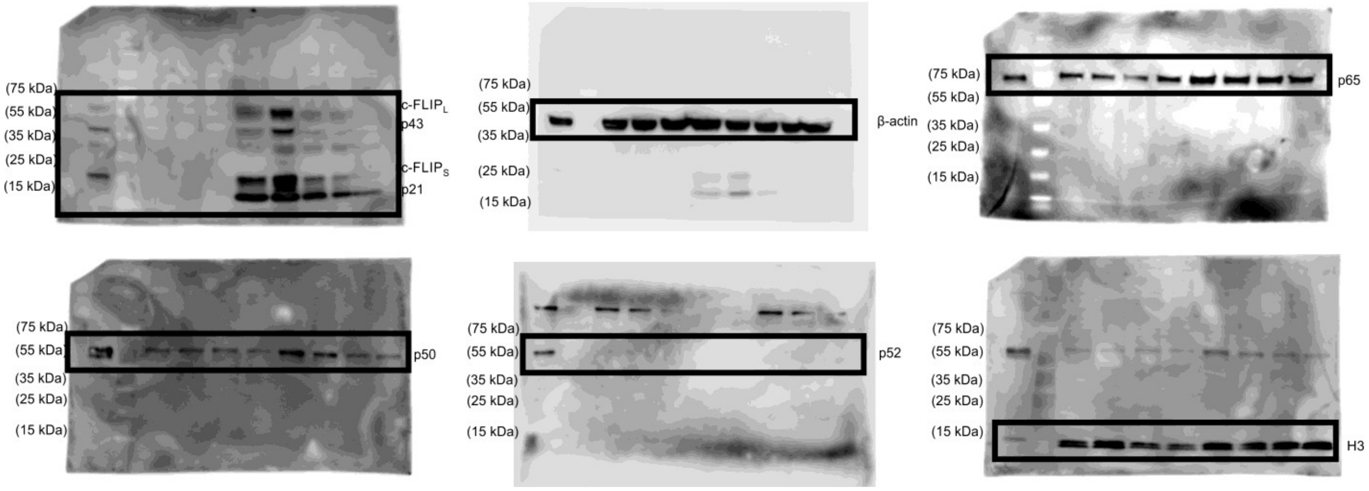

Supplementary Figure 11. Uncropped images of immunoblots displayed in the figures.

Supplementary Table 1

Compliance with REporting recommendations for tumour MARKer prognostic studies (REMARK) guidelines.

**Introduction**	
	Markers analysed are c-FLIP^{+}, PDL-1^{+} and PDL-2^{+} monocytes and plasma level of IL-6.<br><br>Objectives: to select patient with poor prognosis and high risk of early recurrence who may avoid an unnecessary resection.<br><br>Hypothesis: frequency of c-FLIP^{+} monocytes could predict recurrence of pancreatic adenocarcinoma and affect patients' prognosis.
**Materials and Methods**	
- Patients	Treatment naive patients with resectable pancreatic adenocarcinoma.
- Specimen characteristics	Preoperative PBMCs samples.
- Assay method	FLIP protein expression was evaluated by flow cytometry by indirect amplification on intracellular signal. In details,after surface markers stainings, 10^6 PBMCs were fixed and permealized with Foxp3 / Transcription Factor Staining Buffer Set (eBioscience, Thermo Fisher Scientific, Waltham, MA, USA) and purified anti FLIP antibody (D16A8) Cell Signaling Technologies (Danvers, MA, USA) was added for 2 hours at 4°C. Signal was amplified with a secondary anti-rabbit biotin-conjugated antibody (RG-16, 1:1,500) Sigma-Aldrich (Saint Louis, MO, USA) and with Fluorochrome-conjugated Streptavidin eBioscience (Thermo Fisher Scientific, Waltham, MA, USA) both for 30 min at 4°C. Data of IL-6 level in PDAC plasma are obtained from Piro et al., 2017.
- Study design	Prospective study. PBMCs samples collected between 2012 and 2014.<br><br>Clinical end-point of disease free survival (DFS) and overall survival (OS) used.
- Statistical methods	Optimal cut-off thresholds for monocytes frequency assessed by the maximisation of the Youden's statistics.<br><br>Survival curves of patients stratified according to c-FLIP^{+}, PDL-1^{+} and PDL-2^{+} monocytes frequency plus IL-6 level drawn by Kaplan-Meier estimates and compared by log rank test.
**Results**	
- Data	Patient's characteristics in Supplementary Table 2.
- Analysis and presentation	Kaplan–Meier survival curves for effect of FLIP, PDL-1 and PDL-2 expression and IL-6 level on disease free survival and overall survival in Figures 6 and supplementary Figure 10.
**Discussion**	
	Frequency of c-FLIP^{+}PDL1^{+} monocytes and c-FLIP^{+}PDL1^{+}IL-6^{high} are the strongest independent prognostic marker of OS and DFS of pancreatic adenocarcinoma.

Supplementray Table 2

Characteristics of patients involved in the study.

Patients Characteristics	*N°*	%
**Age (years)**		
Median	63	
Range	38-77	
**Gender**		
Female, n (%)	33	47
Male, n (%)	38	53
**Tumor Stage**		
T1, n (%)	1	1.4
T2, n (%)	2	2.8
T3, n (%)	67	94.4
T4, n (%)	1	1.4
**Nodal stage**		
N0, n (%)	10	14
N+, n (%)	61	86
**Metastasis stage**		
M0, n (%)	71	100
M+, n (%)	0	0
**Location**		
head, n (%)	55	77
body/tail, n (%)	16	23
**Resection margins**		
R0, n (%)	28	39
R1, n (%)	43	61
**Adjuvant therapy**		
no, n (%)	10	14
yes, n (%)	61	86
non-gemcitabine based, n (%)	2	3
gemcitabine-based, n (%)	59	97
**Radiotherapy**		
no, n (%)	49	69
yes, n (%)	22	31
**Tumor grade**		
G1, n (%)	5	7
G2, n (%)	46	65
G3, n (%)	20	28

R1 resection denotes a microscopically positive margin; T Tumor; N node; G grade.

Supplementary Table 3

Multivariate analysis of factors influencing OS and DFS in patients with resectable pancreatic cancer.

	multivariate analysis for OS			multivariate analysis for DFS		
**Variable**	**HR**	**(95% CI)**	***P***	**HR**	**(95% CI)**	***P***
**Tumor grade**						
G1-2						
G3	3.349	(1.316 - 8.521)	0.011	2.263	(1.189 - 4.307)	0.013
**$FLIP^+$ $PDL1^+$ $CD14^+$ cells**						
low						
high	2.239	(0.827 - 1.316)	0.113	2.863	(1.117 - 7.342)	0.029

	multivariate analysis for OS			multivariate analysis for DFS		
**Variable**	**HR**	**(95% CI)**	***P***	**HR**	**(95% CI)**	***P***
**Tumor grade**						
G1-2						
G3	3.844	(1.534 - 9.629)	0.004	2.651	(1.381 - 5.092)	0.003
**IL6 / $FLIP^+$ $PDL1^+$ $CD14^+$ cells**						
low						
high	4.225	(1.484 - 12.03)	0.007	2.494	(1.168 - 5.323)	0.018

HR hazard ratio; *CI* confidential interval.
